# Protective effect of platinum nano-antioxidant and nitric oxide against hepatic ischemia-reperfusion injury

**DOI:** 10.1038/s41467-022-29772-w

**Published:** 2022-05-06

**Authors:** Jing Mu, Chunxiao Li, Yu Shi, Guoyong Liu, Jianhua Zou, Dong-Yang Zhang, Chao Jiang, Xiuli Wang, Liangcan He, Peng Huang, Yuxin Yin, Xiaoyuan Chen

**Affiliations:** 1 Institute of Precision Medicine, Peking University Shenzhen Hospital, Shenzhen, 518036 China; 2 Institute of Photomedicine, Shanghai Skin Disease Hospital, School of Medicine, Tongji University, Shanghai, 200443 China; 3 Departments of Diagnostic Radiology, Surgery, Chemical and Biomolecular Engineering, and Biomedical Engineering, Yong Loo Lin School of Medicine and Faculty of Engineering, National University of Singapore, Singapore, 119074 Singapore; 4 Clinical Imaging Research Centre, Centre for Translational Medicine, Yong Loo Lin School of Medicine, National University of Singapore, Singapore, 117599 Singapore; 5 Nanomedicine Translational Research Program, NUS Center for Nanomedicine, Yong Loo Lin School of Medicine, National University of Singapore, Singapore, 117597 Singapore; 6 Marshall Laboratory of Biomedical Engineering, International Cancer Center, Laboratory of Evolutionary Theranostics (LET), School of Biomedical Engineering, Health Science Center, Shenzhen University, Shenzhen, 518060 China; 7 School of Medicine and Health, Harbin Institute of Technology, Harbin, 150001 China

**Keywords:** Preclinical research, Nanomedicine, Biomaterials, Animal disease models, Biomedical engineering

## Abstract

Therapeutic interventions of hepatic ischemia-reperfusion injury to attenuate liver dysfunction or multiple organ failure following liver surgery and transplantation remain limited. Here we present an innovative strategy by integrating a platinum nanoantioxidant and inducible nitric oxide synthase into the zeolitic imidazolate framework-8 based hybrid nanoreactor for effective prevention of ischemia-reperfusion injury. We show that platinum nanoantioxidant can scavenge excessive reactive oxygen species at the injury site and meanwhile generate oxygen for subsequent synthesis of nitric oxide under the catalysis of nitric oxide synthase. We find that such cascade reaction successfully achieves dual protection for the liver through reactive oxygen species clearance and nitric oxide regulation, enabling reduction of oxidative stress, inhibition of macrophage activation and neutrophil recruitment, and ensuring suppression of proinflammatory cytokines. The current work establishes a proof of concept of multifunctional nanotherapeutics against ischemia-reperfusion injury, which may provide a promising intervention solution in clinical use.

## Abstract (web summary)

Pharmacological interventions against hepatic ischemia-reperfusion injury remain limited. Here, the authors provide a nanotherapeutics-based solution combining reactive oxygen species scavenging and nitric oxide modulation.

## Introduction

Currently, liver resection and transplantation have been widely used in the clinic to treat various liver diseases such as intrahepatic bile duct stones, liver trauma, tumors and other diseases^1^. During the surgery, liver ischemia reperfusion injury (IRI) occurs due to the cessation and restoration of blood supply, which may result in an acute inflammatory response, severe liver damage, and even multiple organ failure and death^2–4^. Current intervention strategies for hepatic IRI include ischemic preconditioning, pharmacological agents preconditioning, gene therapy, and so on^5,6^. However, due to the complicated pathophysiological processes of IRI, there is so far no effective solution for the prevention and intervention of IRI in the clinic.

Hepatic IRI is a pathological process involving multiple factors, including acidosis, oxidative stress, intracellular calcium overload, and activation of macrophages and neutrophils caused by hypoxic metabolism^2,7^. Pharmacological interventions such as supplementation of antioxidants to reduce the oxidative stress have been explored to minimize the risk of liver damage^8–10^. The FDA approved drug N-acetylcysteine (NAC) is an antioxidant and glutathione inducer indicated for the treatment of fulminant liver failure due to paracetamol overdose^11^. NAC has also been utilized for ameliorating hepatocyte damage in IRI experimental models, while more clinic outcome data is required before the routine use of NAC^12,13^. Recently, accumulating evidence reveals that nitric oxide (NO) plays diverse roles in modulating cell behaviors and NO-releasing materials have been designed for potential therapeutic applications^14–18^. In particular, it has been reported that the administration of inhaled NO, nitrite or NO donor drugs could attenuate ischemia/reperfusion injury during the liver surgery and accelerate the restoration of liver function^19–21^. However, challenges such as short half-life of NO, ambiguous therapeutic effect and drug tolerance remain and further improvements are needed^17,22–24^.

Since the discovery of Fe_3_O_4_ nanoparticles as the peroxidase mimics^25^, a variety of nanomaterials capable of mimicking the functions of natural enzymes, namely nanozymes, have attracted considerable interest^26–28^. Owing to advantages in stability, low cost, and recyclability, nanozymes have been widely used in areas of chemistry, biology, and medicine^29–32^. Among these nanozymes, several nanomaterials have displayed promising effect on the elimination of reactive oxygen species (ROS), which could be employed as biomimetic antioxidants to regulate ROS homeostasis^31,33–36^. In particular, platinum nanoparticles (Pt NPs) as the substitute of natural enzymes for the treatment of oxidative stress-related diseases have gained growing interest, owing to their cytocompatibility and enzyme-like catalytic ability^37–39^. Pt NPs display the catalase (CAT)-like properties that decompose hydrogen peroxide (H_2_O_2_) into H_2_O and oxygen (O_2_). It was also reported they could scavenge superoxide anion (•O_2_^−^), similar to superoxide dismutase (SOD)^40^. In addition, nanozyme-catalyzed cascade reactions are intriguing for versatile biomedical applications^41–45^. For example, Li *et al*. integrated the artificial Au nanoparticle (NP) nanozyme and natural ATP synthase into hollow silica microspheres for mitochondria-mimicking oxidative phosphorylation^41^. In the designed natural-artificial hybrid architecture, the Au NPs could convert glucose into gluconic acid in the presence of oxygen (O_2_) and the resulting transmembrane proton gradient facilitated the production of ATP catalyzed by ATP synthase. Nanozyme-involved cascade reactions exhibit great benefits in reducing the diffusion barriers, minimizing intermediate decomposition and enhancing local concentrations of reactants, thereby improving the intercommunication and efficiency of catalytic reactions^46–48^.

**Fig. 1:**
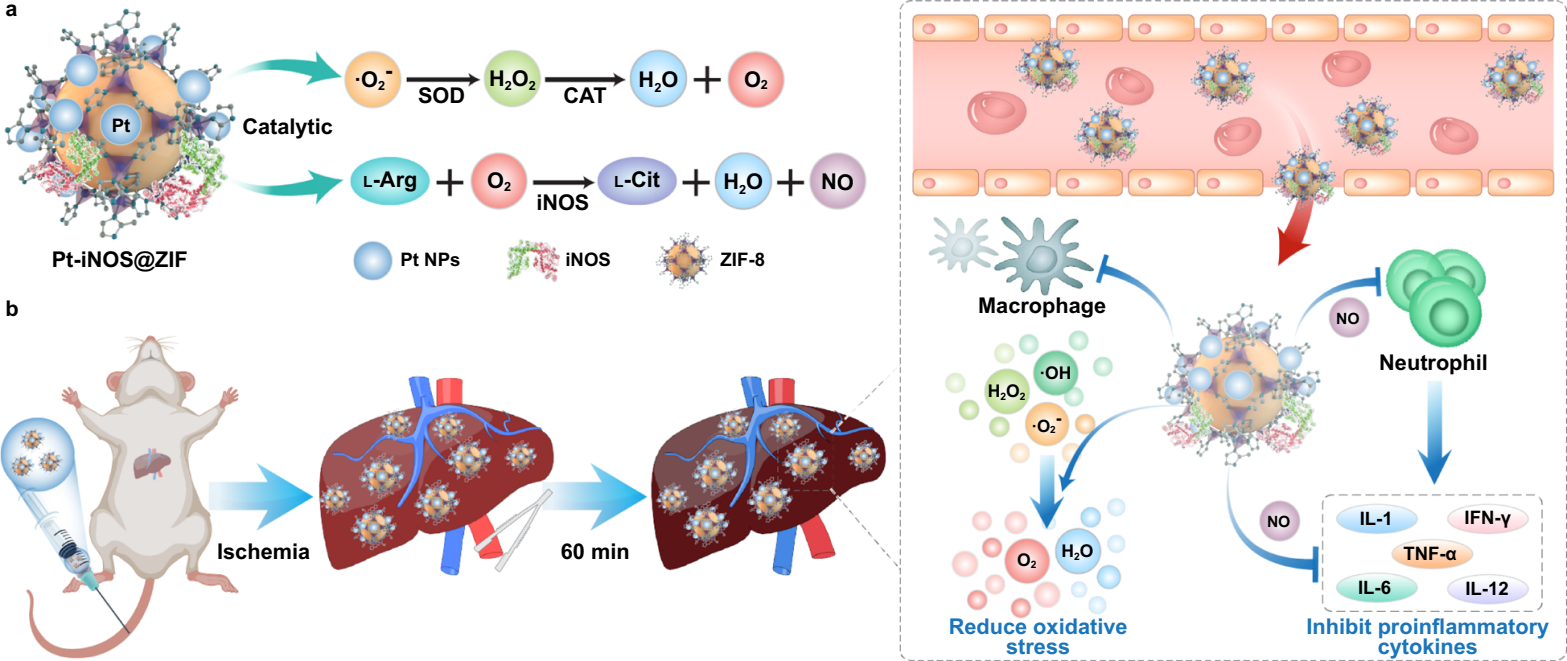
Schematic illustration of hepatic IRI prevention performance by Pt-iNOS@ZIF nanoreactor. **a** Illustration of the designed Pt-iNOS@ZIF nanoreactor. The synthesized Pt nanozyme with SOD/CAT-like properties could scavenge overexpressed ROS induced by IRI to generate O_2_. Then iNOS enzyme could further catalyze ʟ-Arginine (ʟ-Arg) and O_2_. to produce ʟ-Citrulline (ʟ-Cit) and NO. **b** Schematic of the hepatic IRI generation and treatment with the Pt-iNOS@ZIF nanoreactor. It can reduce oxidative stress and inhibit proinflammatory cytokines, resulting in effective prevention the liver from IRI.

In this work, we rationally design a nanozyme-containing biomimetic cascade system to achieve simultaneous NO generation and noxious ROS depletion for effective prevention of IRI, given the endogenous synthesis of NO produced from ʟ-Arginine (ʟ-Arg) by the catalysis of nitric oxide synthases (NOS). To make full use of the inherent advantages of nanozymes and natural enzymes, ultrasmall platinum nanoparticles (Pt NPs) with SOD/CAT-like properties and induced nitric oxide synthase (iNOS) were integrated into the zeolitic imidazolate framework-8 (ZIF-8) carriers to form a safe and effective nanoreactor (Pt-iNOS@ZIF). The process of eliminating ROS by Pt NPs nanozyme generated oxygen, which further promoted the production of NO via the catalysis of iNOS in the presence of ʟ-Arg (Fig. 1a). In the biological context, Pt-iNOS@ZIF was found to greatly reduce oxidative stress-induced damage, inhibit cell apoptosis, and reduce the expression of proinflammatory cytokines, leading to effective intervention of hepatic IRI (Fig. 1b). Overall, the designed nanoreactor not only improves the targeting and bioavailability of NO, but also exhibits dual protective effects via ROS elimination and NO modulation, thereby offering a promising strategy to protect the liver from IRI.

## Results

### Design, synthesis, and characterization of Pt-iNOS@ZIF

**Fig. 2:**
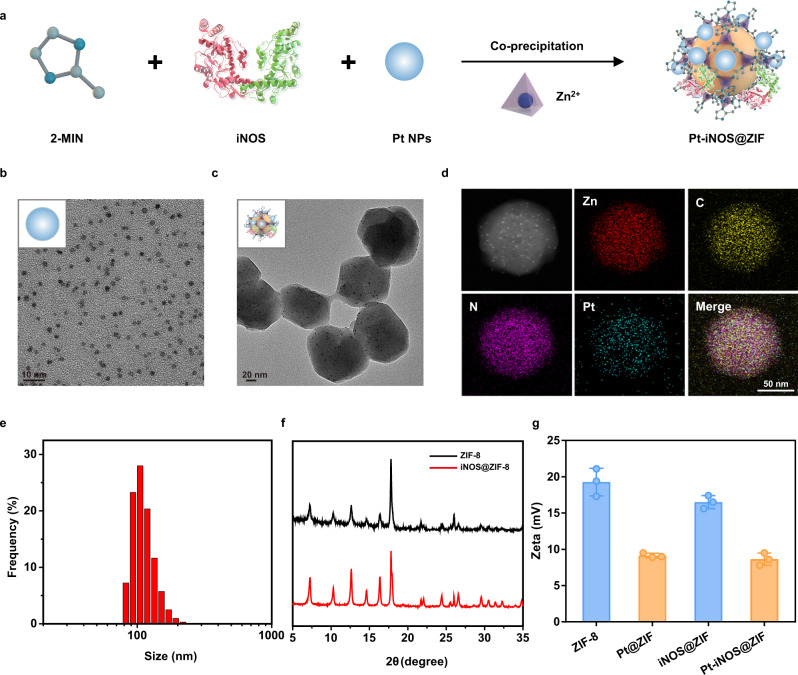
Preparation and characterizations of Pt-iNOS@ZIF nanoreactor. **a** Synthetic procedure of Pt-iNOS@ZIF nanoreactor. **b**, **c** TEM images of the synthesized Pt NPs and Pt-iNOS@ZIF nanoreactor. **d**, **e** Elemental mapping and size distribution of Pt-iNOS@ZIF nanoreactor analyzed by dynamic laser scattering (DLS), scale bar: 50 nm. **f** X-ray diffraction (XRD) patterns of ZIF-8 and iNOS@ZIF-8 NPs. **g** Zeta potentials of different NPs (ZIF-8, Pt@ZIF, iNOS@ZIF and Pt-iNOS@ZIF) (*n* = 3 independent samples). Experiments were performed twice (**d**) or three times (**b**, **c**), with similar results. Data presented as means ± s.d.

In this study, the natural-artificial hybrid nanoreactor was designed and synthesized. First, ultrasmall polyvinylpyrrolidone-coated Pt NPs were successfully synthesized according to the reported method^49^. Then the co-precipitation approach was applied to simultaneously embed enzyme molecules (iNOS) and Pt NPs into the ZIF-8 supporting matrix (Fig. 2a). Transmission electron microscopy (TEM) and elemental mapping results clearly indicated that the obtained Pt NPs had an average size of ~3 nm with a narrow size distribution. Interestingly, the Pt NPs could be effectively deposited in the whole ZIF-8 NPs during the co-precipitation process (Fig. 2b–d). The as-prepared Pt-iNOS@ZIF displayed an average size distribution of 99.7 ± 9.0 nm by dynamic light scattering (DLS) measurement (Fig. 2e). Next, TEM results suggested the loading content of Pt element gradually increased with the increasing feeding amount of Pt NPs (Pt:Zn ratios of 1:250, 1:50, and 1:10) (Supplementary Fig. S1). The optimal loading content of Pt in Pt-iNOS@ZIF with good dispersibility in aqueous solutions was calculated to be 2.3% (wt%) by inductively coupled plasma-optical emission spectrometry (ICP-OES). To investigate the process of enzyme embedding into ZIF-8, the iNOS@ZIF NPs were synthesized, which displayed similar morphology with ZIF-8 NPs. X-ray diffraction (XRD) pattern suggested no significant difference in the crystal structure before and after the enzyme encapsulation (Fig. 2f and Supplementary Fig. S2). In addition, Fig. 2g showed that there were close zeta potential values before and after the iNOS encapsulation, further validating the enzyme embedding, rather than surface absorption processes^50^. In the absence of iNOS, the ZIF-8 NPs had an average size of around 69 ± 8 nm. With the addition of iNOS, the size of enzyme-encapsulated NPs gradually increased, which is likely attributed to the protein-mediated aggregative growth. However, continuous addition of iNOS may result in the increased NP size along with the polydispersity and aggregation (Supplementary Fig. S3). Therefore, we fixed the loading content at around 6.8% with a relatively uniform size less than 100 nm, which is beneficial for cell internalization further in vivo applications. Additionally, the stability of Pt-iNOS@ZIF under biological conditions were carefully evaluated by monitoring the morphology, size and cargo release (Pt and iNOS), respectively. As shown in Supplementary Fig. S4, TEM results indicated that the nanoparticles maintained the integrity at 24 h, while the hydrodynamic diameter gradually increased over time and reach a plateau after incubation for about 6 h. The increase in size was likely attributed to the complexation between proteins in serum and zinc ions from the surface of the composite. Due to the pH-responsive nature of ZIF-8 structure, substantial release of both iNOS (77.3%) and Pt (54.2%) was observed under acidic conditions (pH 6.5) (Supplementary Fig. S5).

### In vitro performance

**Fig. 3:**
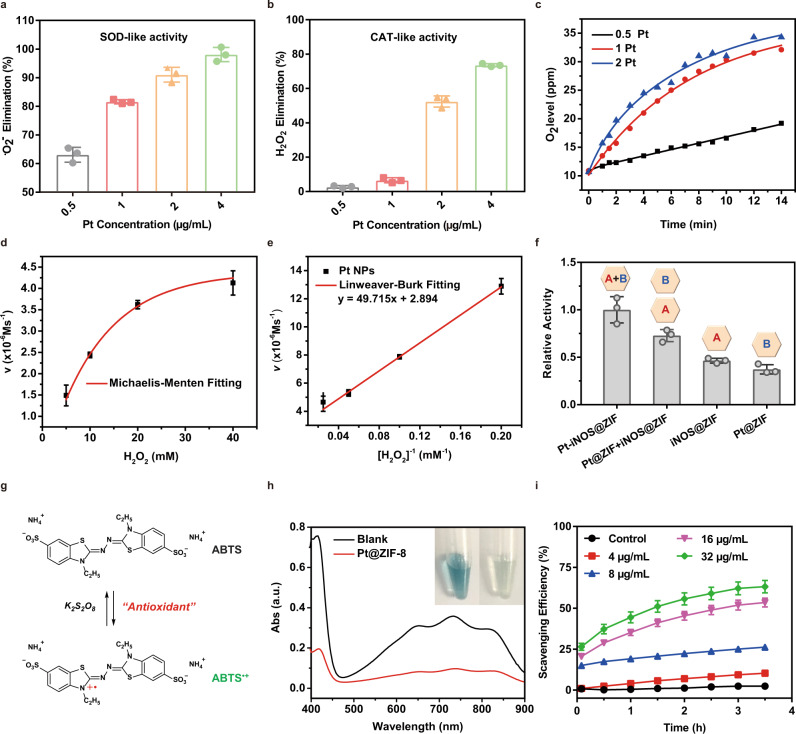
In vitro catalytic performance. **a**, **b** ROS scavenging activities of Pt NPs with SOD-like and CAT-like properties. **c** Concentration-dependent generation of O_2_ in the presence of Pt nanozyme at different concentrations (0.5, 1.0 and 2.0 µg/mL). **d**, **e** Michaelis-Menten steady-state kinetics of O_2_ generation from the decomposition of H_2_O_2_ by Pt NPs. **f** The normalized enzymatic activities of Pt-iNOS@ZIF, Pt@ZIF + iNOS@ZIF, iNOS@ZIF and Pt@ZIF at 12 h time point. The inside symbol [A] = iNOS, [B] = Pt. **g** The antioxidant principle of the ABTS assay. **h** Absorbance and pictures of ABTS radicals with or without treatment of Pt-iNOS@ZIF at 3 h time point. **i** Analysis of the scavenging efficiency of ABTS radicals by different concentrations of Pt-iNOS@ZIF. For **3a**, **b**, **d**–**f**, **i**, *n* = 3 independent samples, data presented as means ± s.d.

Firstly, the enzyme-like catalytic activities of Pt NPs were examined by superoxide dismutase (SOD) activity assay kit and hydrogen peroxide assay kit, respectively. SOD is known to catalyze the superoxide anion (•O_2_^−^) to generate oxygen (O_2_) and hydrogen peroxide (H_2_O_2_), while catalase (CAT) is able to decompose H_2_O_2_ into H_2_O and O_2_. By following the commercially available standard protocols, the inhibition rate of •O_2_^−^ and H_2_O_2_ was obtained. Results demonstrated the elimination ability of both •O_2_^−^ and H_2_O_2_ increased with the increase of Pt NPs concentrations (Fig. 3a, b). To further verify the SOD/CAT mimic capability, the time course of O_2_ generation was monitored by dissolved oxygen probe. In the presence of Pt NPs, the O_2_ level gradually increased over time and the addition of more Pt NPs would undoubtedly accelerate the generation of O_2_ (Fig. 3c). Moreover, the Michaelis-Menten constant (**K**_**M**_) and maximum velocity (**V**_**max**_) were determined to be 172 mM and 3.46 × 10^−6^ M s^−1^, given the Michaelis-Menten curves and Lineweaver-Burk plot (Fig. 3d, e). Taken together, these results clearly demonstrated that Pt NPs exerted both excellent SOD and CAT-like activities, enabling effective elimination of •O_2_^−^ and H_2_O_2._

Given the consumption of O_2_ during the synthesis of NO, we subsequently evaluated whether the generated O_2_ could continuously promote the oxidization of ʟ-Arg into NO. As shown in Fig. 3f, the co-assembled nanoreactor exhibited higher enzymatic activity compared to that of individual enzyme nanoplatform (Pt@ZIF or iNOS@ZIF) or the mixture of two separate systems (Pt@ZIF + iNOS@ZIF) at a fixed time-interval (12 h), clearly demonstrating that the spatial confinement of Pt and iNOS catalyst in the engineered ZIF-8 scaffold enhanced the efficiency of the biocatalytic cascade. To evaluate the overall antioxidant capacity of the as-prepared Pt-iNOS@ZIF nanoreactor, the 2,2′ -azino-bis(3-ethylbenzothiazoline-6-sulfonic acid) (ABTS) assay was performed based on the reduction of ABTS^•+^ radicals by antioxidants (Fig. 3g). As shown in Fig. 3h, the ABTS^•+^ radicals with blue color exhibited obvious absorbance decrease and discoloration in the presence of Pt-iNOS@ZIF. Also, the radical scavenging efficiency is positively correlated with the nanoparticle concentrations, implying favorable antioxidant ability of Pt-iNOS@ZIF to scavenge ATBS radicals (Fig. 3i).

### Cellular evaluation of Pt-iNOS@ZIF

**Fig. 4:**
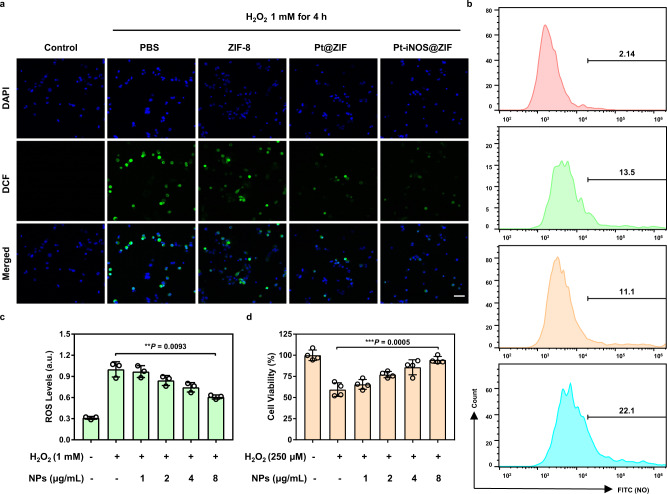
Antioxidant and cell protection activities in FL83B cells. **a** Fluorescence images of ROS levels in H_2_O_2_-stimulated hepatocytes pretreated with PBS, ZIF-8, Pt-ZIF and Pt-iNOS@ZIF NPs (8 μg/mL). Cells were stained with DCFH-DA (green) and Hoechst 33342 (blue) after 4 h incubation. Experiments were performed three times with similar results. Scale bar: 50 μm. **b** Flow cytometry analysis of NO levels with various treatments. The values indicate the percentage of cells staining with NO indicators out of total cells in the selected gate. **c** ROS levels H_2_O_2_-stimulated hepatocytes pretreated with different concentrations of Pt-iNOS@ZIF (0, 1, 2, 4, 8 μg/mL) (n = 3 biologically independent samples). **d** Cell viability in hepatocytes stimulated with H_2_O_2_ for 24 h in the presence of Pt-iNOS@ZIF (0, 1, 2, 4, 8 μg/mL) (n = 4 biologically independent samples). For **c**, **d** data presented as means ± s.d. one-tailed unpaired t-test. ** *P* < 0.01, *** *P* < 0.001.

Next, we examined the antioxidant effects of Pt-iNOS@ZIF in primary mouse hepatocyte FL83B cells. H_2_O_2_ was utilized to stimulate intracellular oxidative stress and 2′,7′-dichlorofluorescein diacetate (DCFH-DA) was applied as ROS indicator. As shown in Fig. 4a, strong green fluorescence signals were observed with the addition of H_2_O_2_ as compared to the control group without H_2_O_2_ treatment. When treated with Pt@ZIF or Pt-iNOS@ZIF, the intracellular fluorescence signals were remarkably decreased, while there was minimal fluorescence disturbance in the ZIF-8 NPs treated group. Quantitative analysis by flow cytometry revealed reduced intracellular ROS level (51.4%) in (Pt@ZIF + H_2_O_2_)-treated cells as compared to that in H_2_O_2_-stimulated cells (59.1%), suggesting the ROS scavenging effect from Pt nanozyme. As comparison, Pt-iNOS@ZIF treated group remarkably decreased ROS level to around 22.8% in H_2_O_2_-pretreated cells (Supplementary Fig. S6). Moreover, the Pt-iNOS@ZIF exhibited concentration-dependent inhibition of ROS generation (Fig. 4c). Subsequently, intracellular NO levels were evaluated by the nitric oxide indicator (DAF-FM diacetate) via flow cytometry analysis and Pt-iNOS@ZIF + H_2_O_2_ group exhibited stronger fluorescence signals than the other groups (Fig. 4b). Then the protective effect of Pt-iNOS@ZIF against H_2_O_2_-induced oxidative stress in cells was evaluated by the standard methyl thiazolyl tetrazolium (MTT) assay. Unsurprisingly, H_2_O_2_ (250 μM) treatment resulted in about 41% cell death after incubation for 24 h, while the addition of Pt-iNOS@ZIF exhibited concentration-dependent increase of the cell viability, implying its protective effect against oxidative damage (Fig. 4d). Compared to the Pt@ZIF or iNOS@ZIF alone, Pt-iNOS@ZIF exhibited improved therapeutic effect. Although simultaneous addition of Pt@ZIF and iNOS@ZIF achieved similar protective effect, the coformulation ensured equal biodistribution, which is valuable for the tandem reaction-based therapy (Supplementary Fig. S7). Moreover, all designed NPs would induce negligible cell death in hepatocytes, HEK 293 cells and macrophage cells (up to 40 µg/mL), suggesting their good biocompatibility and minimum side effect from the nanoformulas themselves (Supplementary Fig. S8-S12). Collectively, all these results demonstrated that Pt-iNOS@ZIF-8 NPs could effectively alleviate oxidative stress and rescue cells from ROS-induced damage.

### In vivo imaging, biodistribution and pharmacokinetics

**Fig. 5:**
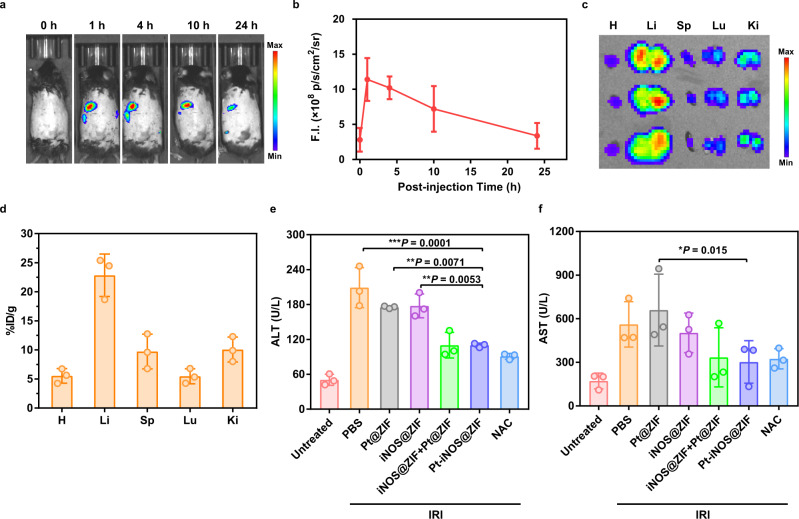
In vivo performance. **a** Representative in vivo fluorescence images of C57BL/6 mice at 1, 4, 10, and 24 h p.i. of Pt-iNOS@ZIF (*n* = 3). **b** Quantitative ROI assays of the fluorescence intensity in mice at designated time points. **c** Ex vivo fluorescence images of major organs at 24 h p.i. H: heart, Li: liver, Sp: spleen, Lu: lung, Ki: kidney. **d** Biodistribution of Pt-iNOS@ZIF in major organs at 24 h p.i. evaluated by ICP-OES. **e**, **f** Serum ALT and AST levels in mice after 60 min of ischemia and 12 h of reperfusion from each group. For 5**b**, **d**–**f**, *n* = 3 biologically independent animals. Data presented as means ± s.d. *P* values were calculated by ANOVA *F*-test, each comparison stands alone. *P < 0.05; **P < 0.01, ***P < 0.001.

In comparison with small molecular drugs, various types of nanomaterials have intrinsic advantages of passive targeting and accumulation in the liver. To evaluate the biodistribution and liver accumulation of Pt-iNOS@ZIF in living systems, Cy5 labeled iNOS enzyme was applied to form Pt-iNOS (Cy5)@ZIF and real-time fluorescence imaging was carried out in mice after intravenous injection (i.v.) of the NPs. As shown in Fig. 5a–c and Supplementary Fig. S13, there was an obvious increase of fluorescence signals at 1 h post injection (p.i.) and semi-quantitative analysis revealed that the NPs could remain in the liver for up to 10 h (7.2 × 10^8^ p/s/cm^2^/sr) and were gradually cleared from the liver by 24 h (3.4 × 10^8^ p/s/cm^2^/sr) p.i. Moreover, the pharmacokinetics and ex vivo biodistribution in major organs at 24 h were verified by ICP-OES. The results demonstrated that the NPs were able to efficiently accumulate in the liver (22.84 ± 3.65%ID g^−1^), serving as an optimal candidate for IRI intervention (Fig. 5d, Supplementary Fig. S14 and Table 1).

### Protective effect in a hepatic IRI model

**Fig. 6:**
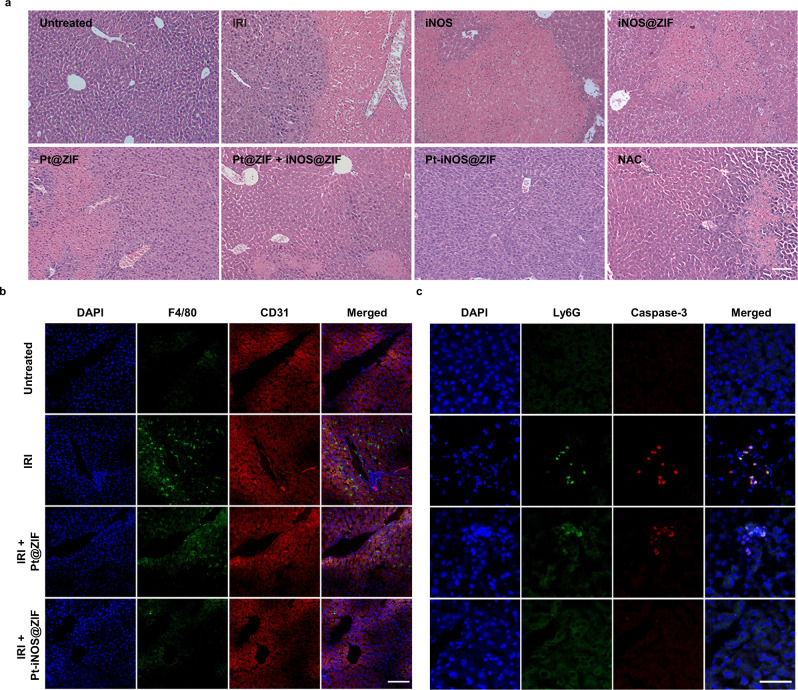
H&E and Immunofluorescence staining on liver tissues. **a** H&E staining of liver tissues from each group (Untreated, PBS, iNOS, iNOS@ZIF, Pt@ZIF, Pt@ZIF+iNOS@ZIF, Pt-iNOS@ZIF, NAC) after 60 min of ischemia and 12 h of reperfusion. Scale bar: 100 µm. **b** Immunofluorescence staining of liver tissues with various treatments by using DAPI (blue) for nuclear staining, anti-F4/80 antibody (green) as monocyte/macrophage marker, and anti-CD31 antibody (red) as an endothelial marker from each group. Scale bar: 100 µm. **c** Enlarged images of immunofluorescence staining with various treatments by using DAPI (blue) for nuclear staining, anti-Ly6G antibody (green) as neutrophil marker and anti-caspase-3 antibody (red) as a cell apoptosis marker. Experiments were performed three times (**a**–**c**) with similar results. Scale bar: 50 µm.

Based on the promising antioxidant effect of Pt-iNOS@ZIF, we further investigated the feasibility of the protective effect against IRI in a murine model. C57BL/6 mice were treated with different nanoplatforms for 12 h before the clamping of porta hepatis. The untreated group underwent the same procedure, but without vascular occlusion. After 60 min of ischemia and subsequent reperfusion, blood and liver samples were collected and liver functions at 12 h after surgery were further analyzed. Alanine aminotransferase (ALT) and aspartate aminotransferase (AST) levels were applied as indicators of liver damage. To better evaluate the therapeutic effect, the antioxidant drug NAC was administered intravenously for comparison before surgery. Compared to the untreated control group, the IRI mice group displayed significant increase of ALT and AST levels, indicating alleviated liver injury. When the IRI mice were pretreated with different types of NPs, the Pt-iNOS@ZIF treated IRI mouse group had remarkably reduced ALT and AST levels compared to those in Pt@ZIF or iNOS@ZIF treated groups. Moreover, Pt-iNOS@ZIF at the dosage of 2 mg/kg achieved comparable therapeutic effect to that of NAC therapy (150 mg/kg), clearly demonstrating its advantage in intervention of liver injury (Fig. 5e–f and Supplementary Fig. S15). To further evaluate the therapeutic efficacy, hematoxylin and eosin (H&E) staining in liver tissues and blood test were performed after pretreatment with various NPs in IRI mice. As expected, severe hepatocyte necrosis and cytolysis was observed in the PBS-treated IRI group after 12 h. The ZIF-8 NPs, Pt NPs and iNOS treated groups displayed indistinguishable difference in the liver injury. iNOS@ZIF and Pt@ZIF slightly attenuated the damage, while it seemed there was no significant difference between the mixture of two separate system (Pt@ZIF + iNOS@ZIF) and co-assembled Pt-iNOS@ZIF. Given the complex physiological processes and multiple regulation factors in IRI, the mechanisms of action will need to be further explored, yet these results do not diminish the importance of coformulation with respect to simple operation and equal biodistribution (Fig. 6a and Supplementary Figs. S16-S17). Overall, the results were consistent with the trend of ALT and AST levels. Besides the therapeutic effectiveness of the designed nanomedicine, the safety in living systems is also important for further potential clinic applications. Hemolysis test (24 h p.i.) and H&E staining of major organs after injection of Pt-iNOS@ZIF for 7 days were evaluated (Supplementary Fig. S18-S21). All results showed Pt-iNOS@ZIF exhibited excellent biocompatibility and could be considered as an ideal candidate for the prevention of IRI.

Liver IRI is a pathophysiological event and oxidative stress is considered as one essential mechanism. To further obtain visual evidence for cellular events, immunofluorescence imaging was performed on liver tissues. Results indicated that hepatic IRI led to obvious activation of monocytes/macrophages compared to the untreated group. The designed Pt@ZIF NPs can slightly inhibit the activation of macrophages, likely due to its ROS scavenging effect (Fig. 6b). By employing both ROS scavenging derived from Pt nanozyme and NO-based modulating effect, Pt-iNOS@ZIF treated mice exhibited minimal activation of monocyte/macrophages. Similarly, the Pt-iNOS@ZIF treated group also greatly suppressed the recruitment of neutrophils and caspase activities for preventing cells from apoptosis compared to the IRI group (Fig. 6c, Supplementary Fig. S22 and Table 2). Taken together, all these findings indicated that the preconditioning of Pt-iNOS@ZIF could inhibits IRI-induced macrophage activation, neutrophil accumulation, and subsequent apoptotic processes.

**Fig. 7:**
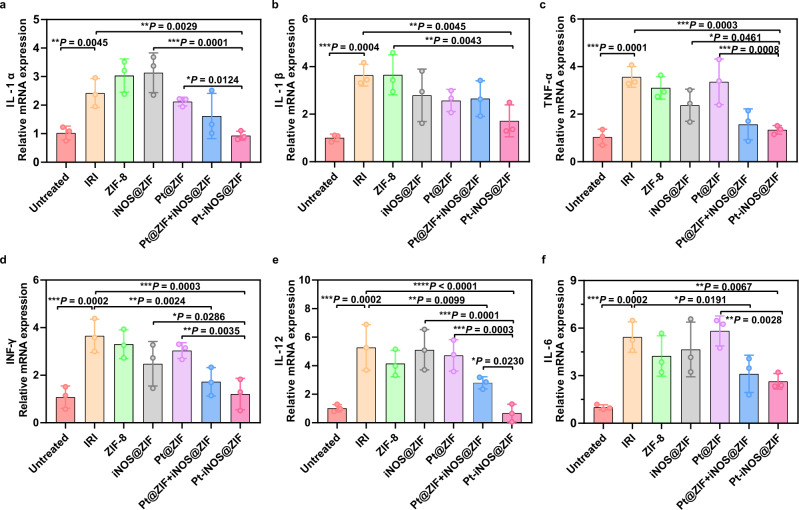
Anti-inflammatory activities in hepatic IRI. Relative expression of mRNAs for cytokines of IL-1α (**a**), IL-1β (**b**), INF-γ (**c**), IL-12α (**d**), TNF-α (**e**), IL-6 (**f**) (*n* = 3 biologically independent animals). Data presented as means ± s.d. from three independent replicates, and *P* values were calculated by ANOVA *F*-test, each comparison stands alone. *P < 0.05; **P < 0.01, ***P < 0.001.

Encouraged by the above results, we further investigated the anti-inflammatory activities of the designed Pt-iNOS@ZIF, the expression of mRNAs for the pro-inflammatory mediators, including interleukin-1 beta (IL-1β), interleukin-1α (IL-1α), interleukin-6 (IL-6), interleukin-12 (IL-12), tumor necrosis factor-α (TNF-α), and interferon gamma (INF-γ) were measured from each group. As shown in Fig. 7, the levels of these pro-inflammatory cytokines were significantly increased in the hepatic IRI group, while they were reduced to relatively normal ranges in Pt-iNOS@ZIF treated IRI group. TNF-α is one intensively studied cytokine in response to inflammatory and immunomodulatory stimuli, and is a regulator responsible for the production of ROS. Also, IL-1 can facilitate the synthesis of TNF-α by Kupffer cells and recruitment of neutrophils^51^. IFN-γ produced mainly by activated natural killer T cells will promote Kupffer cells or dendritic cell activation. Activation of Kupffer cells and neutrophils in turn induces the release of various chemokines and cytokines, including TNF-α, IL-1β, IL-6, IL-12, etc. which further activates local immune cells, recruits circulating immune cells and aggravates liver damage^52^. Taken together, these results indicated that Pt-iNOS@ZIF preconditioning would significantly suppress the expression of proinflammatory cytokines, which is generally involved in the initiation and propagation of IRI.

## Discussion

IRI include a series of complicated processes including the restriction of blood supply, subsequent restoration and reoxygenation, during which the imbalance of metabolic supply, inflammation and oxidative damage are involved. Additionally, the innate and adaptive immune responses will be activated, resulting in cell damage and organ dysfunction^2^. Oxidative stress is considered to play a pivotal role in ischemia and reperfusion damage. It has been reported that ROS scavenging via supplementation of antioxidants has the ability of diminishing oxidative stress and ensuing organ injury^53^. SOD can promote the conversion of •O_2_^−^ to O_2_ and H_2_O_2_. Also, H_2_O_2_ can be decomposed to H_2_O and O_2_ under the catalysis of CAT. Therefore, both enzymes exhibited beneficial actions by accelerating the detoxification of ROS in IRI. However, the short half-life of these natural proteases in the body (half-life of SOD is about 6 min), difficulty of cell uptake, and low delivery efficiency hinder their further applications^8^. Besides, NO, one of therapeutic gaseous molecules, has multiple regulatory functions, such as improving the microcirculation, suppressing caspase activities, and inhibiting neutrophil infiltration and platelet aggregation^54^. NO-based therapy has been applied for pulmonary hypertension and cardiopulmonary disorders for many years^55^, and its therapeutic effect on protection liver from IRI has increasingly received considerable attention^20,56^.

In this study, we successfully synthesized ultrasmall Pt NPs, which could act as SOD and CAT mimics with excellent ROS scavenging capacity. In order to further enhance the therapeutic efficacy, NO-based therapy is incorporated by integrating the iNOS enzyme and Pt nanozyme into ZIF-8 architecture to achieve synergistic effect. Co-precipitation method was applied to obtain the hybrid nanoreactor, ensuring efficient embedding of iNOS into ZIF-8 during the process of nanoparticle formation. Such design not only prevents natural enzymes from inactivation and degradation in the body, but also promotes the molecular diffusion and intercommunication due to the intrinsic porous feature and large surface areas of metal-organic frameworks^57,58^. More importantly, as the synthesis of NO is O_2_-dependent, the generated O_2_ by Pt catalysis will further improve the iNOS-mediated cascade reaction activities. After the ischemia and reperfusion, the restoration of blood flow will cause the release of ROS, activation of macrophage cells and recruitment of neutrophils, which further leads to the release of large amount of chemokines and cytokines^2^. Apart from the ROS elimination by Pt nanozyme, protective actions of NO during ischemia and reperfusion are probably attributed to antioxidant and anti-inflammatory effects. The prepared Pt-iNOS@ZIF nanoreactor could maintain ALT and AST levels of IRI mice in the relatively normal range. Additionally, it greatly suppressed the activation of macrophages cells, along with the minimized infiltration of neutrophils and expression of pro-inflammatory cytokines, which eventually attenuated the liver damage caused by IRI. Considering the pathological process of induction of oxidative stress in various types of IRI, the cytoprotective effects of this approach in the liver may be applicable to other organ systems, such as heart and kidneys. It should be noted that nitric oxide may display both protective and toxic effects, depending on the NO levels, NO source, and timing of administration. Hence the therapeutic window for IRI in different organs should be carefully evaluated.

In summary, we developed an ZIF-8-based hybrid nanoreactor, which encapsulated the Pt nanozyme and natural enzyme iNOS, for protecting the liver from IRI. By using the excessive ROS generated during hepatic ischemia/reperfusion as a stimulus, the Pt nanozyme with SOD/CAT-like properties could scavenge •O_2_^−^ and H_2_O_2_ to produce O_2_, which can further react with ʟ-Arg to form NO under the catalysis of iNOS enzyme. Such designed cascade reaction successfully achieved the ROS scavenging (•O_2_^−^ and H_2_O_2_) and NO production, which effectively reduced oxidative stress and expression of proinflammatory cytokines in the process of hepatic ischemia and reperfusion. So far, the pharmacological agents in routine clinical use to prevent liver IRI are rare, likely due to their short blood circulation time, poor solubility of most antioxidant and anti-inflammatory drugs and non-specific biodistribution after systemic administration. Our designed nanoformulation enables effective therapeutic outcome with a relatively low dosage, which is likely attributed to the improved pharmacokinetics and tissue distribution. More importantly, the intrinsic ROS in the pathological regions as the reactant initiate the catalytic reaction, thus conferring high specificity for mitigating side effects. In-depth understanding of the molecular mechanisms of IRI will be of great importance in improving current treatment regimens and developing innovative intervention strategies. Besides, efforts should also be made on the combination of multiple drugs, which will improve the effectiveness, success of liver surgery and survival rate of patients. Overall, this study may not only provide a promising solution of multifunctional nanotherapeutics for the treatment of IRI, but also shed light on dual protection mechanism of ROS clearance and NO regulation, which is beneficial to advance further clinical applications in hepatic IRI.

## Methods

### Materials

Hexachloroplatinic acid hexahydrate (H_2_PtCl_6_‚ 6H_2_O), polyvinylpyrrolidone (PVP), sodium borohydride (NaBH_4_), 2-methylimidazole (2-MIN), nitric oxide synthases (iNOS) enzyme, ʟ-Arginine, 2’, 7’-dichlorofluorescein diacetate (DCFH-DA), methyl thiazolyl tetrazolium (MTT), 2,2’-Azino-bis(3-Ethylbenzothiazoline-6-Sulfonic Acid) (ABTS), SOD assay kit, and hydrogen peroxide assay kit were purchased from Sigma-Aldrich. The Griess assay kit was purchased from Promega Corporation (U.S.A). Singlet oxygen sensor green (SOSG), Hoechst 33342 solution, and DAF-FM Diacetate were purchased from Thermo Fisher Scientific (Waltham, MA, U.S.A.). The primer pairs for indicated gentes were provided by Shanghai Personal Gene Technology Co., Ltd. China. All other ere purchased from Sigma without further purification.

### Characterizations

Transmission electron microscopy (TEM) images were acquired on the FEI Tecnai T12 electron microscope. Elemental mapping was acquired on the scanning transmission electron microscope (JEM-3200FS, JEOL, Japan). Dynamic light scattering (DLS) measurements were performed at a SZ-100 nano particle analyzer (HORIBA Scientific). The XRD pattern was recorded on a D8 Advance diffractometer (Bruker, Germany), UV-Vis absorption spectrum was recorded on a Shimadzu UV-2501 spectrophotometer. Inductively coupled plasma optical emission spectroscopy (ICP-OES) was used to quantify Zn and Pt concentrations. Fluorescence images were acquired by confocal laser scanning microscopy (Zeiss LSM 780, Carl Zeiss Inc., Jena, Germany). Flow cytometry analysis was performed on a BD Accuri C6 flow cytometry (BD Biosciences) and the data were analyzed using FlowJo V10. Tissue and tumor samples for eosin (H&E) staining were prepared by Shanghai Weiao Biotechnology Co. Ltd (Shanghai, China) and observed using a BX41 bright field microscopy (Olympus).

### Syntheis of platnium nanoparticles (Pt NPs)

The ultra-small platinum nanoparticles were prepared according to the reported method with slight modification^49^: Briefly, in 10 mL of 1 mM hexachloroplatinic acid hexahydrate (H_2_PtCl_6_•6H_2_O) solution, 111.1 mg of polyvinylpyrrolidone (PVP) was added. After stirring for 15 min, 200 µL freshly prepared sodium borohydride (NaBH_4_) solution (100 mM) was added and stirred for 12 h. The solution turned dark brown and Pt NPs were obtained for further use.

### Syntheis of ZIF-8, Pt@ZIF, Pt-iNOS@ZIF Nanosystms

The ZIF-8 nanoparticles were synthesized according to the reported method^50^. Briefly,100 μL Zn(NO_3_)_2_•6H_2_O aqueous solution (0.5 M) was added into 900 μL 2-methylimidazole (2-MIN, 3.5 M) and the mixture was stirred at room temperature for 1 h. The obtained product was collected by centrifugation (3500  × *g*, 10 min) and washed with water for three times. Similarly, nanoscale Pt@ZIF was synthesized by mixing the 50 µL Pt NP solution with 900 μL 2-methylimidazole, followed by the additon of Zn(NO_3_)_2_•6H_2_O, The resulting Pt@ZIF was redispersed in water for further use. To synthezie nanoscale Pt-iNOS@ZIF, 0.3 mg iNOS enzyme was incubated with 900 μL 2-MIN for 10 min at 30 °C followed by addition of zinc nitrate. For the synthesis of Pt-iNOS(Cy5)@ZIF, 0.5 mg cyanine 5 NHS ester (Lumiprobe) was reacted with iNOS (0.3 mg) in PBS solution for overnight. Then the product was dialyzed to remove excessive dye molecules. The obtained iNOS (Cy5) was used to prepare Pt-iNOS(Cy5)@ZIF by using the same method for the synthesis of Pt-iNOS@ZIF. After centrifugation and washing with water for three times, the obtained products were characterized by transmission electron microscopy (TEM) and dynamic light scattering (DLS).

1 $$m=\rho \times \frac{4}{3}{{{{{\rm{\pi }}}}}}{{{{{{\rm{r}}}}}}}^{3}$$

2 $$m\left({{{{{\rm{Pt}}}}}}\right){{\mbox{total}}}=\omega \left({{{{{\rm{Pt}}}}}}\right)\times \frac{m\left({{{{{\rm{ZIF}}}}}}\right)}{\omega \left({{{{{\rm{ZIF}}}}}}\right)}$$

3 $$N=\frac{m\left({{{{{\rm{Pt}}}}}}\right){{\mbox{total}}}}{m\left({{{{{\rm{Pt}}}}}}\right)\,{{\mbox{per}}}\,{{\mbox{particle}}}}=\frac{m\left({{{{{\rm{Pt}}}}}}\right){{\mbox{total}}}}{\rho \left({{{{{\rm{Pt}}}}}}\right)\times \frac{4}{3}{{{{{\rm{\pi }}}}}}{{{{{{\rm{r}}}}}}({{{{{\rm{Pt}}}}}})}^{3}}=41{{{{{\rm{partiles}}}}}}$$

In the Eqs. 1–3, $$\rho ({{{{{\rm{ZIF}}}}}})=0.95\,{{{{{\rm{g}}}}}}\,{{{{{{\rm{cm}}}}}}}^{-3}$$$${{\mbox{,}}}$$ $$\,\rho ({{{{{\rm{Pt}}}}}})=21.45\,{{{{{\rm{g}}}}}}\,{{{{{{\rm{cm}}}}}}}^{-3}$$ ; *ω*(Pt) is the loading content of Pt

NPs, *ω*(ZIF) is the loading content of ZIF-8 NPs. *m*(Pt)_total_ is the total weight of Pt element in one Pt-iNOS@ZIF particle. The average number of Pt NPs in one Pt-iNOS@ZIF was calculated to be about 41.

### Assays of SOD-like and CAT-like activities

To evaluate the Pt nanozyme properties, the superoxide anion scavenging activity was conducted with a SOD assay kit (Sigma-Aldrich, USA). The hydrogen peroxide quenching activity was performed with the Amplex® red hydrogen peroxide assay kit (Sigma-Aldrich, USA). All expreiments were carried out according to the standard protocol. To monitor the O_2_ production, different concentrations of Pt NPs (0.5, 1.0 and 2.0 µg/mL) were mixed with 10 mM H_2_O_2_ in PBS buffer (pH 7.4), the dissiolved oxygen levels over time were measured by a dissolved oxygen probe. To obtain the enzyme kinetic parameters, different concencentraions of H_2_O_2_ were incubated with Pt NPs (2.0 µg/mL) for 10 min, the Michaelis-Menten constant (**K**_**M**_) and maximum velocity (**V**_**max**_) were determined as following equation: $v=\frac{{{{{{{\rm{V}}}}}}}_{{\max }}{{{{{\rm{\cdot }}}}}}[{{{{{\rm{S}}}}}}]}{\left[{{{{{\rm{S}}}}}}\right]+{{{{{{\rm{K}}}}}}}_{m}}$.

### In vitro measurement of total antioxidant capacity

The ABTS^•+^ radical scavenging assay was applied to evaluate the total antioxidant capacity of Pt-iNOS@ZIF. Briefly, 7 mM ABTS in deionized water was prepared and reacted with 2.45 mM potassium persulfate overnight. Then the solution turned dark blue and ABTS radical cation (ABTS^•+^) was obtained. Next, 200 µL diluted solution was mixed with different concentrations of Pt-iNOS@ZIF for 5 min and the UV-Vis spectra were recorded. The radical scavenging efficiency was calculated based on the absorbance changes at 734 nm. The data were analyzed using Origin 2015 software

### General methods for cell culture

The murine hepatocyte FL83B cells (ATCC, #CRL-2390) were grown in F12K media supplemented with 10% fetal bovine serum (FBS, Gibico), 100 U/ml penicillin, and 100 mg/ml streptomycin at 5% CO_2_ and 37 °C. The human embryonic kidney 293 cells (HEK 293) (ATCC, #CRL-1573), Raw264.7 cells (ATCC, TIB-71), Kupffer cells (Thermal Fisher Scientific, RTKCCS) were grown in Dulbecco’s modified eagle medium (DMEM, Gibico) supplemented with 10% FBS at 5% CO_2_ and 37 °C.

### Intracellular ROS and NO measurements

Intracellular ROS levels were measured using 2’,7’-dichlorofluorescein diacetate (DCFH-DA) as the ROS indicator and NO levles were measured using nitric oxide indicators (DAF FM Diacetate). 8 μg/mL different types of NPs (ZIF-8, Pt@ZIF, Pt-iNOS@ZIF) were added into cells and cultured for 2 h, followed by the treatment with H_2_O_2_ (1 mM) and incubated for another 4 h at 37 °C. Then, the cells were stained with DCFH-DA (10 μM) or DAF probe (5 μM) for 30 min. After removing the excessive probe, fluoresence images were acquired by confocal laser scanning microscopy (CLSM). H33342 channel (λ_ex_ = 408 nm), FITC channel (λ_ex_ = 488 nm). Quantative ROS levels with dfferent concentrations of Pt-iNOS@ZIF treatment (1, 2, 4, 8 µg/mL) were analyzed by flow cytometry.

### Cell viability assay

FL83B and HEK 293 cells were seeded in 96-well plates with a density of 1.0 × 10^4^ cells per well. After 24 h incubation, the fresh medium containing different concentrations of Pt-iNOS@ZIF (1, 2, 4, 8 µg/mL) were added and incubated for another 2 h before addition of H_2_O_2_ (250 μM). Then cells were incubated for 24 h at 37 °C under 5% CO_2_ and the cytotoxicity was evaluated by the standard MTT assay. The absorbance at 570 nm was measured by the microplate reader.

### Preparation of hepatic IRI model in mice

Male C57BL/6 mice (6–8 weeks old) were received from Zhejiang Experimental Animal Center and were fed with a standard diet and water. The animals were hosted in equipped animal facility with ambient temperature of 22 °C and humidity at 30–70%, under a dark/light cycle of 12 h. All animal laboratory operations were carried out according to the Guide of Animal Ethics Committee of Shanghai Skin Disease Hospital. For hepatic IRI model preparation, the mice were fasted for 12 h before the surgical operation. After the mice were anesthetized with isoflurane, they were placed on a heated surgical pad and the abdomen of the depilated mice was disinfected with iodophor solution. Then a midline laparotomy was performed to expose the portal triplet. The portal triplet was carefully lifted using a vessel forceps, and all structures in the portal triplet (hepatic artery, portal vein, and bile duct) were blocked using a microvascular clamp. The abdominal wall was covered with PBS-soaked gauze and the blocking process lasted 60 min. After 60 min, microvascular clamp was removed for reperfusion. Signs of the reperfusion can be observed by the immediate color change of the central lobe and the left lobe of the liver.

### In vivo imaging and biodistribution

For the fluorescence imaging, 100 μL Cy5-labled nanocomposite (Pt-iNOS(Cy5)@ZIF, 2 mg/kg) solutions was intravenously injected into male C57BL/6 mice (6–8 weeks old). In vivo fluorescence imaging was recorded at 1, 4, 10 and 24 h p.i. on an IVIS Spectrum system. At 24 h post injection, the above mice were sacrificed, and major organs were collected and washed before optical imaging. The fluorescence intensity of livers was acquired from the analysis of the region of interest (ROI) using a Living Image software. Moreover, the ex vivo biodistribution in major organs at 24 h was also measured by ICP-OES. The organs were weighted, and the percentage of injected dose per gram (%ID g^−1^) of tissue were calculated as the following equation: $\% {{{{{\rm{ID}}}}}}/{{{{{{\rm{g}}}}}}}^{-1}=\frac{{{{{{\rm{Dose}}}}}}\,{{{{{\rm{in}}}}}}\,{{{{{\rm{Organ}}}}}}}{{{{{{\rm{Injected}}}}}}\,{{{{{\rm{Dose}}}}}}\times {{{{{\rm{Organ}}}}}}\,{{{{{\rm{Weight}}}}}}}$. The total injected does is 100 µg/mL. For pharmacokinetic studies, Pt-iNOS@ZIF was intravenously injected into mice and the blood samples were collected at 0.5, 1, 4, 12, and 24 h. Then blood samples were centrifuged at 10000 x g for 10 min to obtain the plasma. Then the supernatant was digested by aqua regia and the amount of Pt was quantified by ICP-MS.

### Protective effect in a hepatic IRI model

The mice were randomly divided into six groups (*n* = 5) different formulations: (1) Untreated, (2) PBS + IRI, (3) ZIF-8 + IRI, (4) Pt NPs + IRI, (5) Pt@ZIF + IRI, (6) Pt-iNOS@ZIF + IRI. Nanoformulations (2 mg/kg) in each group were intravenously injected 12 h before surgical operation. After 12 h induction of the hepatic IRI model in mice, the blood was collected for biochemical analysis. Liver and kidney functions were evaluated by blood tests of alanine aminotransferase (ALT) levels and aspartate aminotransferase (AST) levels (Shanghai Institute of Materia Medica, Center of Drug Safety Evaluation Research, CDSER, SIMM). The left liver lobes were dissected for hematoxylin and eosin (H&E) and immunofluorescence staining.

### Immunofluorescence staining

Frozen tissue sections were prepared and covered with OCT media. Then the liver tissue sections were fixed with zinc fixative solutions for 10 min. After rinsing slides with PBS for 3 times, the liver tissues were further treated with 2% Triton X-100 for 15 min. Then 100 µL blocking buffer (10% FBS in PBS) was added onto tissues for 1 h. Antibodies including F4/80 Monoclonal Antibody (BM8), FITC (Thermo Fisher Scientific, #11-4801-85, 1:100), Alexa Fluor® 594 anti-mouse CD31 Antibody (Biolegend, #102520, 1:100), anti-mouse Ly6G/Ly6C (Gr-1) (BioXcel, #BE0075, 1:200) and Anti-Caspase-3 antibody (Abcam, #ab13847, 1:200) were diluted and added on slides and incubated for overnight at 4 °C. To stain the secondary antibody, slides were washed and incubated with secondary antibody conjugated with Goat Anti-Rabbit IgG H&L (Alexa Fluor 594) (Abcam, #ab150080, 1:200) or Goat Anti-Rat IgG H&L (Alexa Fluor 488) (Abcam, #ab150157, 1:200) for 1 h. After that, the slides were rinsed with PBS and mounted with DAPI-containing mounting solution. Fluorescence images were acquired via confocal microscopy.

### Biocompatibility analysis

To evaluate the biocompatibility of nanoparticles, liver and kidney functions were assessed in healthy mice after intravenous injection of Pt-iNOS@ZIF for 7 days. Liver and kidney functions of untreated mice were also evaluated as control. Major organs (heart, liver, spleen, lung, and kidney) were harvested at 24 h and 7 days for hematoxylin and eosin (H&E) staining. H&E staining slides were viewed using a BX41 bright field microscope (Olympus).

### Measurement of inflammatory cytokine levels in liver tissues

To evaluate inflammatory cytokine levels,the left liver lobe tissues in each group were harvested at 12 h after surgery to detect the relative mRNA expression of IL-1α, IL-1β, TNF-α, IFN-γ, IL-12 and IL-6 using quantitative real-time polymerase chain reaction (qPCR) assay. Total RNA was extracted from the obtained liver tissues and reverse transcripted into cDNA in a gradient RNA apparatus. Subsequently, fluorescence qPCR amplification was performed and the relative mRNA expression levels of the cytokines mentioned above were measured and calculated. The primer pairs for indicated gentes were described in Supplementary Table 3.

### Statistical analysis

Quantitative data were presented as mean ± s.d. Statistical differences were calculated by Student’s t-test using Excel 2019 software and A One-way analysis of the variance (ANOVA) using GraphPad Prism 7.0 software when appropriate. *P* values < 0.05 were considered statistically significant and illustrated by *P < 0.05, **P < 0.01, ***P < 0.001, respectively.

### Reporting summary

Further information on research design is available in the Nature Research Reporting Summary linked to this article.

## Supplementary information

Supplementary information

Supplementary figure

Supplementary Data files

Reporting Summary

## Data Availability

The authors declare that the experimental data supporting the findings of this study are available within the article and the Supplementary Information Files. Extra data are available corresponding author upon reasonable request. Source data are provided with this paper.
