# Supplementary material for: Protective effect of platinum nano-antioxidant and nitric oxide against hepatic ischemia-reperfusion injury
Source: Nat Commun. 2022 May 6;13:2513. doi: 10.1038/s41467-022-29772-w (PMC9076604; doi:10.1038/s41467-022-29772-w)
Supplement: Supplementary file 1 — Supplementary information [file 41467_2022_29772_MOESM1_ESM.docx]

***Supplementary Information***

**Protective Effect of Platinum Nano-antioxidant and Nitric Oxide Against Hepatic Ischemia-Reperfusion Injury**

Jing Mu^1†^, Chunxiao Li^2†^, Yu Shi^1^, Guoyong Liu^1^, Jianhua Zou^3,4,5^, Dong-Yang Zhang ^6^, Chao Jiang^6^, Xiuli Wang^2^, Liangcan He^7^*, Peng Huang^6^*, Yuxin Yin^1^*, Xiaoyuan Chen^3,4,5^*

^1^Institute of Precision Medicine, Peking University Shenzhen Hospital, Shenzhen, 518036, China.

^2^Institute of Photomedicine, Shanghai Skin Disease Hospital, School of Medicine, Tongji University, Shanghai 200092, P. R. China.

^3^Departments of Diagnostic Radiology, Surgery, Chemical and Biomolecular Engineering, and Biomedical Engineering, Yong Loo Lin School of Medicine and Faculty of Engineering, National University of Singapore, Singapore, 119074, Singapore.

^4^Clinical Imaging Research Centre, Centre for Translational Medicine, Yong Loo Lin School of Medicine, National University of Singapore, Singapore 117599, Singapore

^5^Nanomedicine Translational Research Program, NUS Center for Nanomedicine, Yong Loo Lin School of Medicine, National University of Singapore, Singapore 117597, Singapore

^6^Marshall Laboratory of Biomedical Engineering, International Cancer Center, Laboratory of Evolutionary Theranostics (LET), School of Biomedical Engineering, Health Science Center, Shenzhen University, Shenzhen, 518060, China.

^7^School of Medicine and Health, Harbin Institute of Technology, Harbin 150080, China.

†These authors contributed equally to this work.

Email: [liangcanhe@hit.edu.cn](mailto:liangcanhe@hit.edu.cn) (L. H); [peng.huang@szu.edu.cn](mailto:peng.huang@szu.edu.cn) (P. H); [yinyuxin@bjmu.edu.cn (Y](mailto:yinyuxin@bjmu.edu.cn%20(Y). Y); [chen.shawn@nus.edu.sg](mailto:chen.shawn@nus.edu.sg) (X.C.)

This PDF file includes

**Supplementary Fig. S1 TEM images of Pt@ZIF NPs with different Pt loading concentrations**........S5

**Supplementary Fig. S2 TEM images of ZIF-8 NPs**…….……...........................…………...…………S6

**Supplementary Fig. S3** **TEM images of** **iNOS@ZIF NPs with different iNOS loading concentrations**…………………………………………………………………………...……………...S7

**Supplementary Fig. S4** **The stability of Pt-iNOS@ZIF in biological conditions**.………….................S8

**Supplementary Fig. S5 The release of Pt-iNOS@ZIF under different pH conditions**.……...............S9

**Supplementary Fig. S6 Flow cytometry analysis of intracellular ROS levels**....................................S10

**Supplementary Fig. S7 Cell viability assay treated with indicated NPs in H_2_O_2_-stimuliated cells**...S11

**Supplementary Fig. S8 Cell viability treated with various concentrations of Pt NPs**........................S12

**Supplementary Fig. S9 Cell viability treated with indicated NPs in hepatocytes**..............................S13

**Supplementary Fig. S10 Cell viability treated with indicated NPs in HEK 293 cells**........................S14

**Supplementary Fig. S11 Cell viability treated with indicated NPs in Raw264.7 cells**……………...S15

**Supplementary Fig. S12 Cell viability treated with indicated NPs in Kupffer cells**..........................S16

**Supplementary Fig. S13 In vivo fluorescence imaging**……….…………...…………………………S17

**Supplementary Fig. S14 Pharmacokinetics assay of Pt-iNOS@ZIF in mice**……………………… S18

**Supplementary Fig. S15 Serum ALT and AST levels with various treatments**.....….……………...S19

**Supplementary Fig. S16 Representative H&E staining and quantification with indicated treatments after 12 h of reperfusion**.........................................................................................................................S20

**Supplementary Fig. S17 Blood test for the evaluation of treatment effect**….....................................S21

**Supplementary Fig. S18 Hemolysis test of Pt-iNOS@ZIF**...........................................................…...S22

**Supplementary Fig. S19** **Profiles of liver functions after injection with Pt-iNOS@ZIF for 7 days**..S23

**Supplementary Fig. S20 Profiles of kidney functions after injection with Pt-iNOS@ZIF for 7 days**….…………………………………………………………………………………………………S24

**Supplementary Fig. S21 H&E staining images of major organs in healthy mice**…......….………...S25

**Supplementary Fig. S22 Representative immunofluorescence staining on liver tissues**…...............S26

**Supplementary Table 1. The biodistribution analysis in major organs** …........................................S27

**Supplementary Table 2. Quantification of immunofluorescence staining in Fig. 6c**.........................S27

**Supplementary Table 3. Primer information for mouse**.....................................................................S28


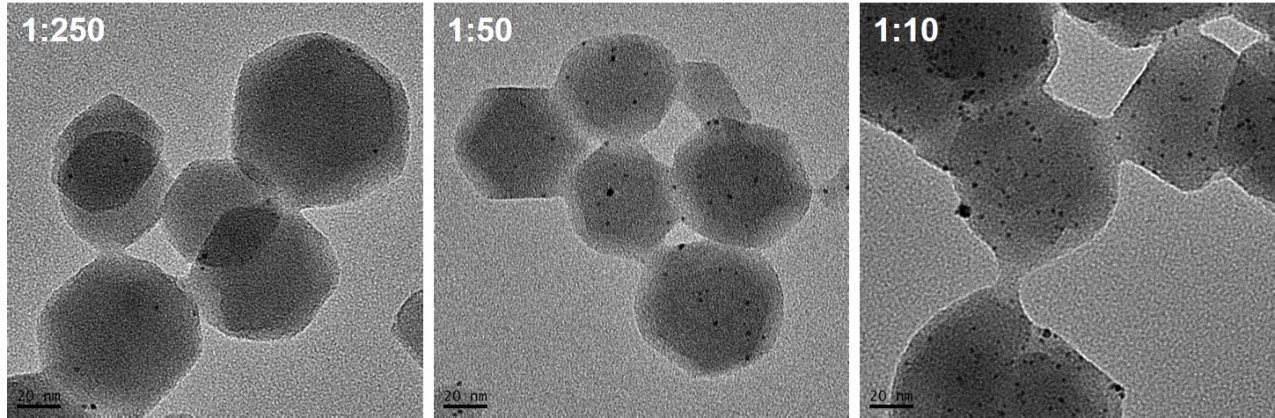


**Supplementary Fig. S1 TEM images of Pt@ZIF NPs with different Pt loading concentrations.** The loading content of Pt element gradually increased with the increase of the feeding amount of Pt NPs (Pt: Zn ratios of 1:250, 1:50, 1:10). Their average sizes are 69 ± 12 nm, 67 ± 7 nm, 70 ± 8 nm, respectively. Experiments were performed three times with similar results.


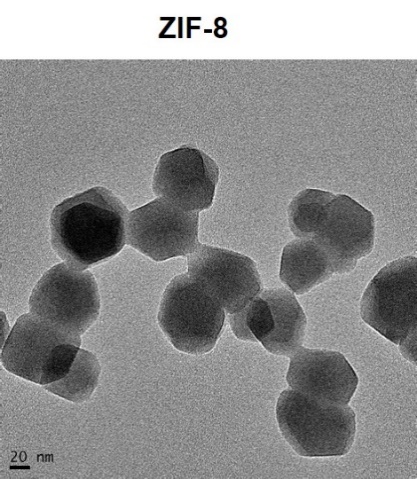


**Supplementary Fig. S2 TEM images of ZIF-8 NPs.** Experiments were performed three times with similar results


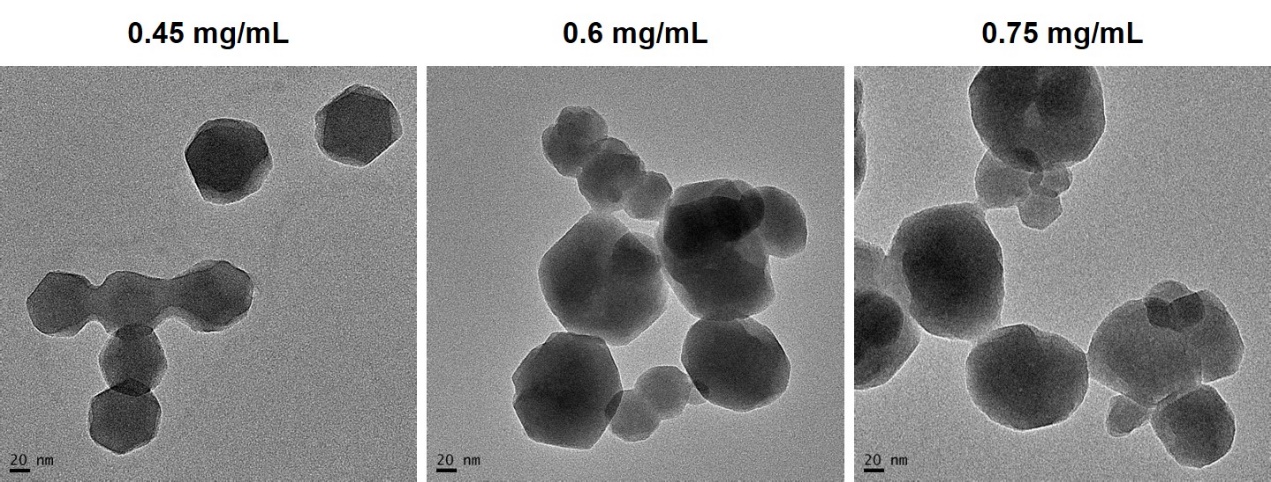


**Supplementary Fig. S3 TEM images of iNOS@ZIF NPs with different iNOS loading concentrations.** Different amount of iNOS (0.45, 0.6 and 0.75 mg/mL) was added during the preparation of iNOS@ZIF. Their average sizes were 77 ± 7 nm, 95 ± 32 nm, 106 ± 42 nm, respectively. The images indicated that increasing iNOS amount resulted in increased size of NPs along with the polydispersity and aggregation. Experiments were performed three times with similar results.


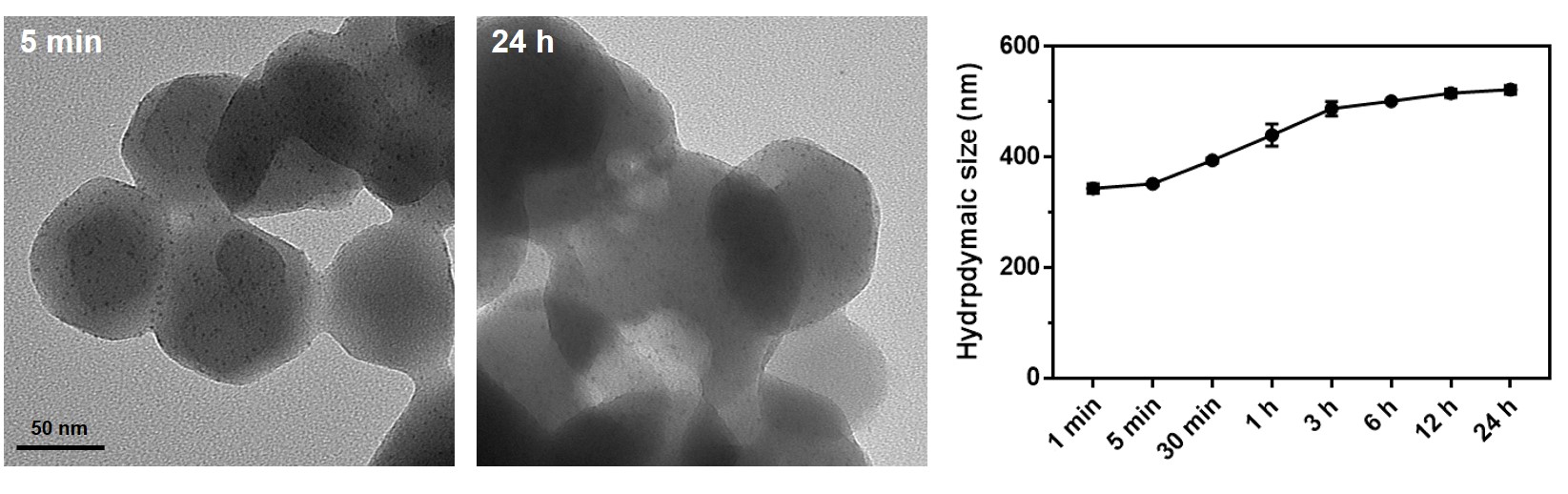


**Supplementary Fig. S4 The stability of Pt-iNOS@ZIF in biological medium.** The stability of Pt-iNOS@ZIF was tested in HEPES buffer with 10% FBS. The integrity and hydrodynamic size of particles were characterized by TEM and DLS (n = 3 independent samples). Experiments were performed three times with similar results. Data are presented as means ± s.d.


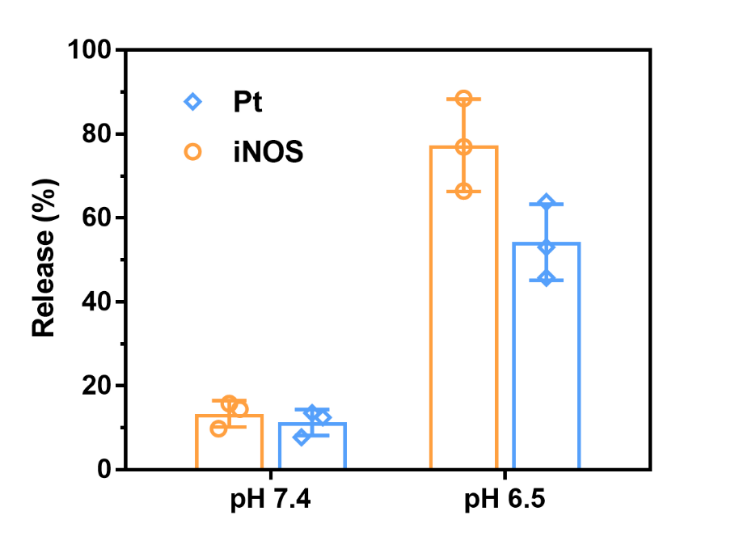


**Supplementary Fig. S5** **The release of Pt-iNOS@ZIF under different pH conditions.** The release of Pt and iNOS in HEPES buffer with 10% FBS under different pH conditions was tested (n = 3 independent samples). The ICP and BCA protein assay are used to monitor the release of Pt and iNOS, respectively. Data are presented as means ± s.d.

.


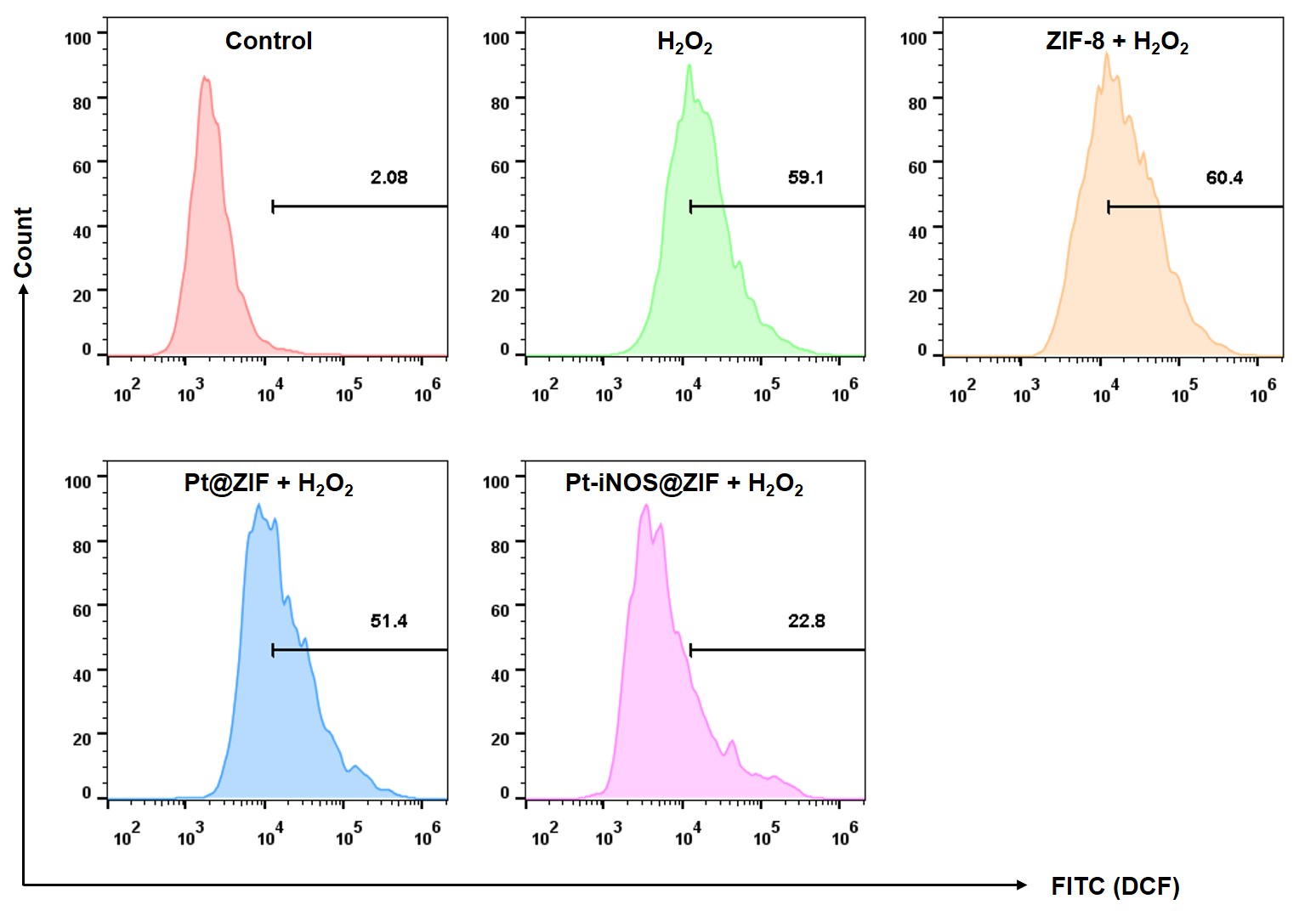


**Supplementary Fig. S6 Flow cytometry analysis of intracellular ROS levels.** The Fluorescence images of ROS levels in H_2_O_2_-stimulated hepatocytes pretreated with PBS, ZIF-8, Pt@ZIF and Pt-iNOS@ZIF (8 μg/mL). Cells were stained with DCFH-DA (green) after 4 h incubation and analyzed by flow cytometry.


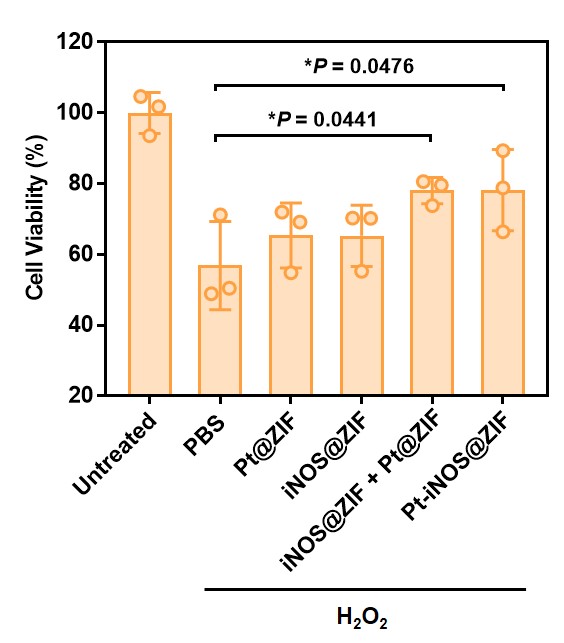


**Supplementary Fig. S7 Cell viability assay treated with indicated NPs in H_2_O_2_-stimuliated cells**. Hepatocytes were stimulated with H_2_O_2_ incubated with Pt@ZIF, iNOS@ZIF, Pt@ZIF+iNOS@ZIF or Pt-iNOS@ZIF (4 μg/mL) for 24 h before the viability measurements by MTT assays (n = 3 biologically independent samples, one-tailed unpaired t-test). Data represents mean ± s.d. * *P* < 0.05.


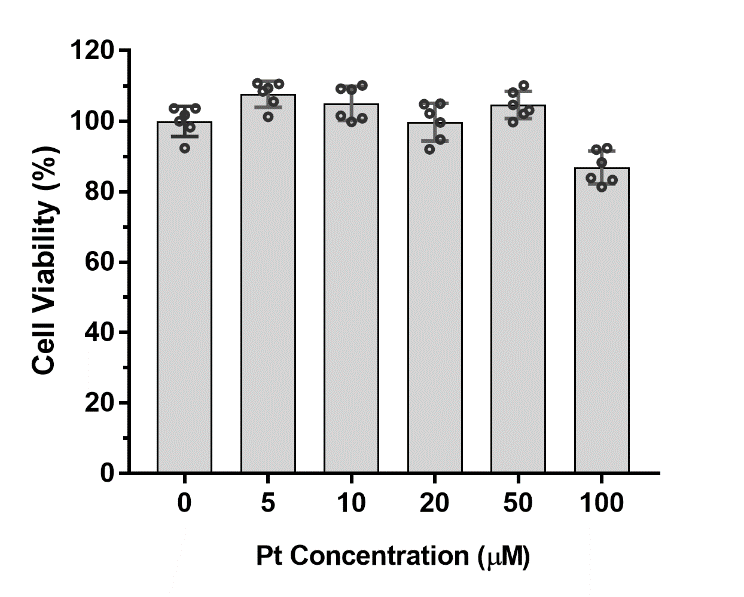


**Supplementary Fig. S****8 Cell viability treated with various concentrations of Pt NPs.** The Pt NPs were incubated with HEK 293 cells for 48 h before the viability measurements by MTT assays (n = 6 biologically independent samples). Data represents mean ± s.d.


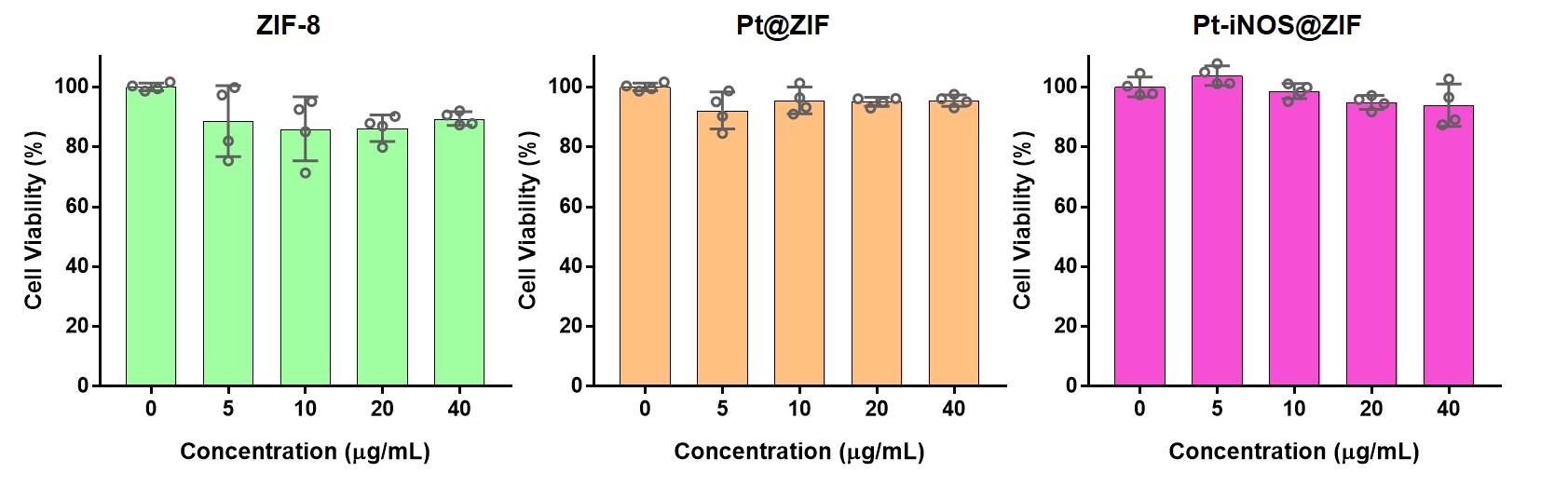


**Supplementary Fig. S9 Cell viability treated with indicated NPs in hepatocytes.** The ZIF-8, Pt@ZIF and Pt-iNOS@ZIF NPs were incubated with FL83B cells for 48 h before the viability measurements by MTT assays (n = 4 biologically independent samples). Data represents mean ± s.d.


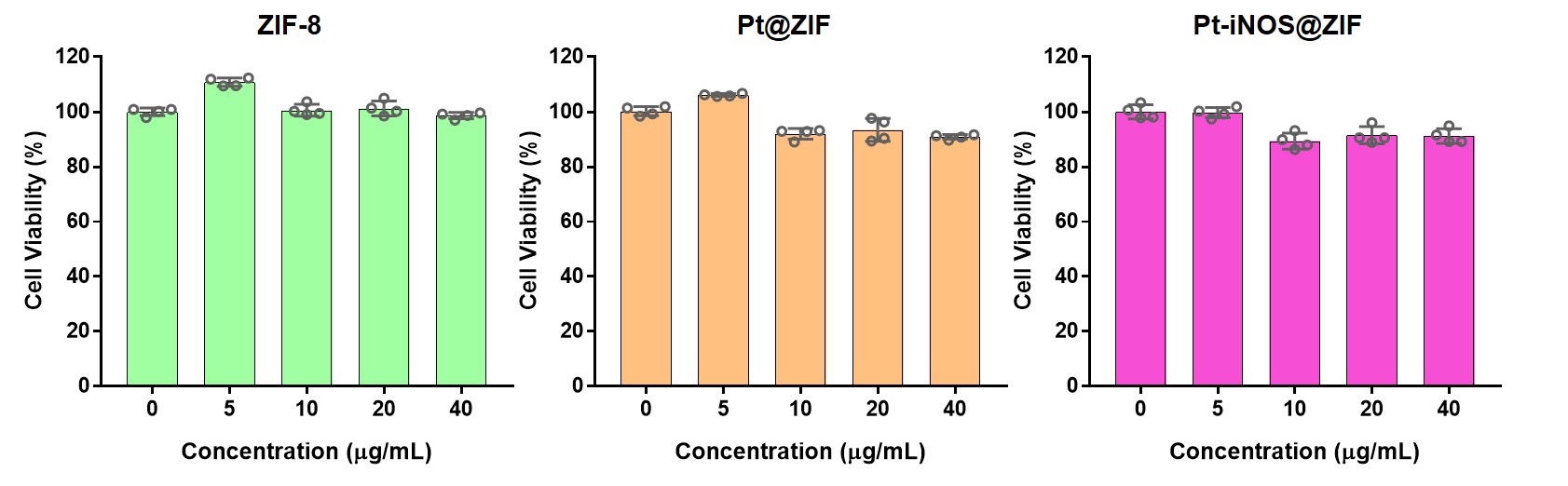


**Supplementary Fig. S10 Cell viability treated with indicated NPs in HEK 293 cells.** The ZIF-8, Pt@ZIF and Pt-iNOS@ZIF NPs were incubated with HEK 293 cells for 48 h before the viability measurements by MTT assays (n = 4 biologically independent samples). Data represents mean ± s.d.


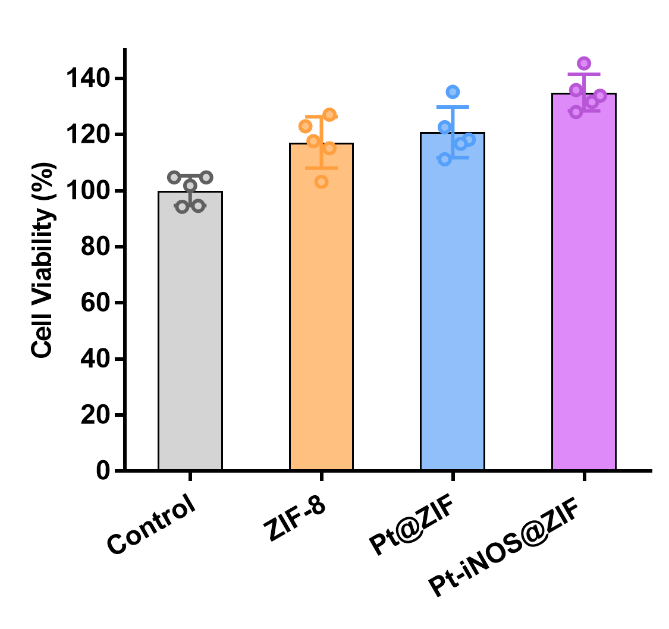


**Supplementary Fig. S11 Cell viability treated with indicated NPs in Raw264.7 cells.** The ZIF-8, Pt@ZIF and Pt-iNOS@ZIF NPs were incubated with Raw264.7 cells for 48 h before the viability measurements by MTT assays (n = 5 biologically independent samples). Data represents mean ± s.d.


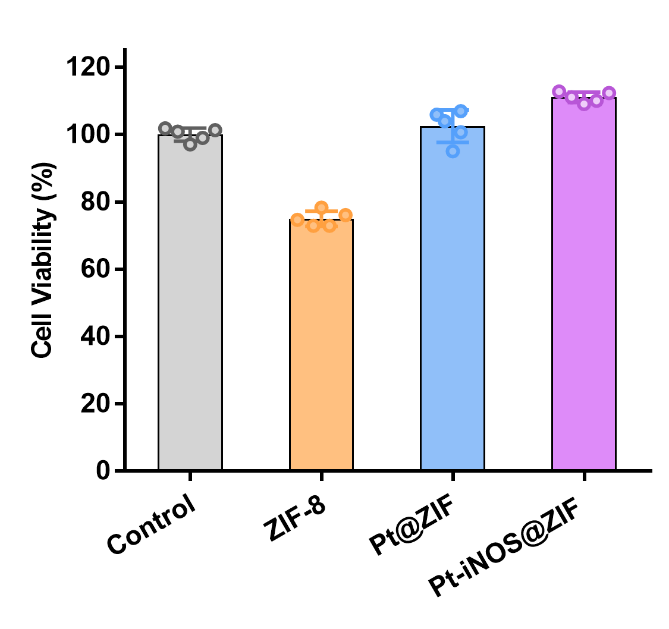


**Supplementary Fig. S12 Cell viability treated with indicated NPs in Kupffer cells.** The ZIF-8, Pt@ZIF and Pt-iNOS@ZIF NPs were incubated with Kupffer cells for 48 h before the viability measurements by MTT assays (n = 5 biologically independent samples). Data represents mean ± s.d.

**Supplementary Fig. S13 In vivo fluorescence imaging.** Representative *in vivo* fluorescence images of C57BL/6 mice at 1, 4, 10 and 24 h p.i. of Pt-iNOS(Cy5)@ZIF (n = 3 biologically independent animals) performed on an IVIS Spectrum system.


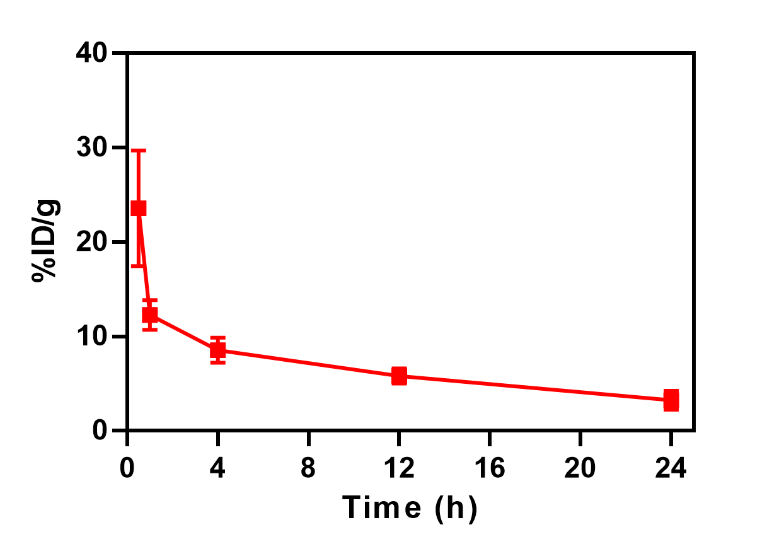


**Supplementary Fig. S14** **Pharmacokinetics of Pt-iNOS@ZIF in mice after intravenous injection** (n = 3 biologically independent animals)**.** (*t*_1/2_ = 0.56 h)**.** Data are presented as means ± s.d.


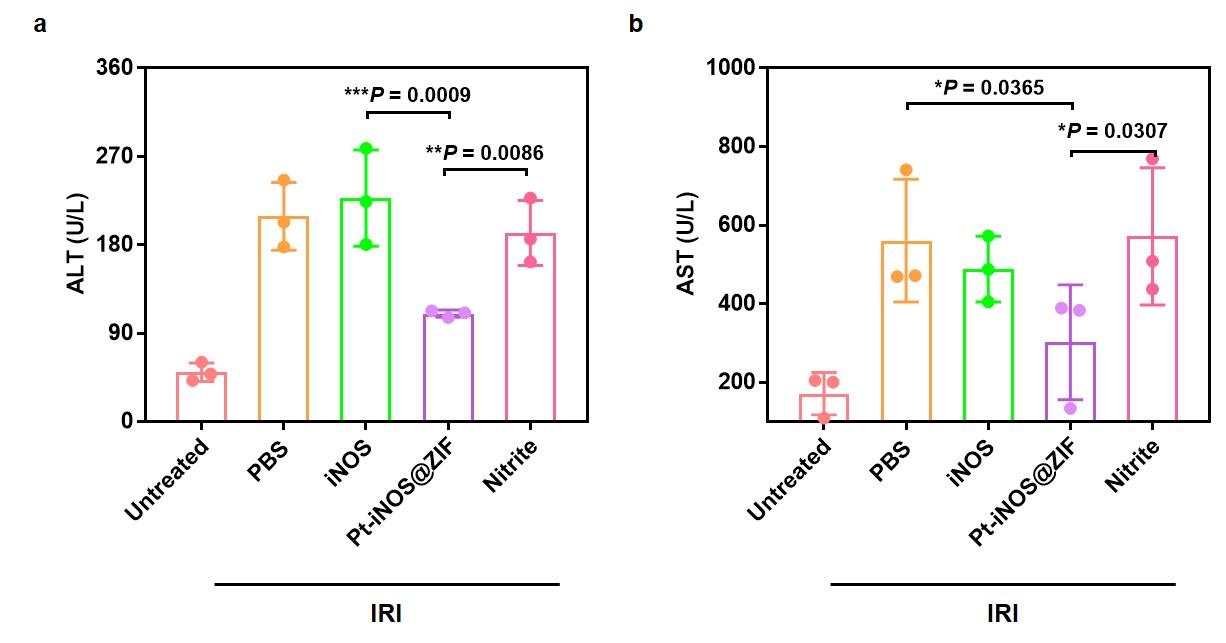


**Supplementary Fig. S15 Serum ALT and AST levels in mice with various treatments.** Different formulations: (1) untreated, (2) PBS + IRI, (3) iNOS+ IRI, (4) Pt-iNOS@ZIF + IRI, (5) Nitrite (480 nmol) + IRI. Nanoformulations in each group were intravenously injected into mice 12 h before surgical operation. sodium nitrite was administered intraperitoneally two minutes before the surgery. Sodium nitrite was administered intraperitoneally two minutes before the surgery. After 12 h induction of the hepatic IRI model in mice, the blood was collected for biochemical analysis. n = 3, biologically independent animals. Data presented as means ± s.d. ANOVA *F*-test. Each comparison stands alone. * *P* < 0.05; ** *P* < 0.01, *** *P* < 0.001

**Supplementary Fig. S16 Representative H&E staining and quantification with indicated treatments after 12h of reperfusion.** (a) H&E staining of liver tissues from each group (untreated, ZIF-8, Pt NPs) after 60 min of ischemia and 12 h of reperfusion. Experiments were performed three times with similar results. Scale bar: 100 µm. (b) Quantification of the relative IR area from H&E staining images using Image J Software (ANOVA *F*-test, each comparison stands alone). n = 3, biologically independent animals. Data presented as means ± s.d. * *P* < 0.05; ** *P* < 0.01, *** *P* < 0.001.


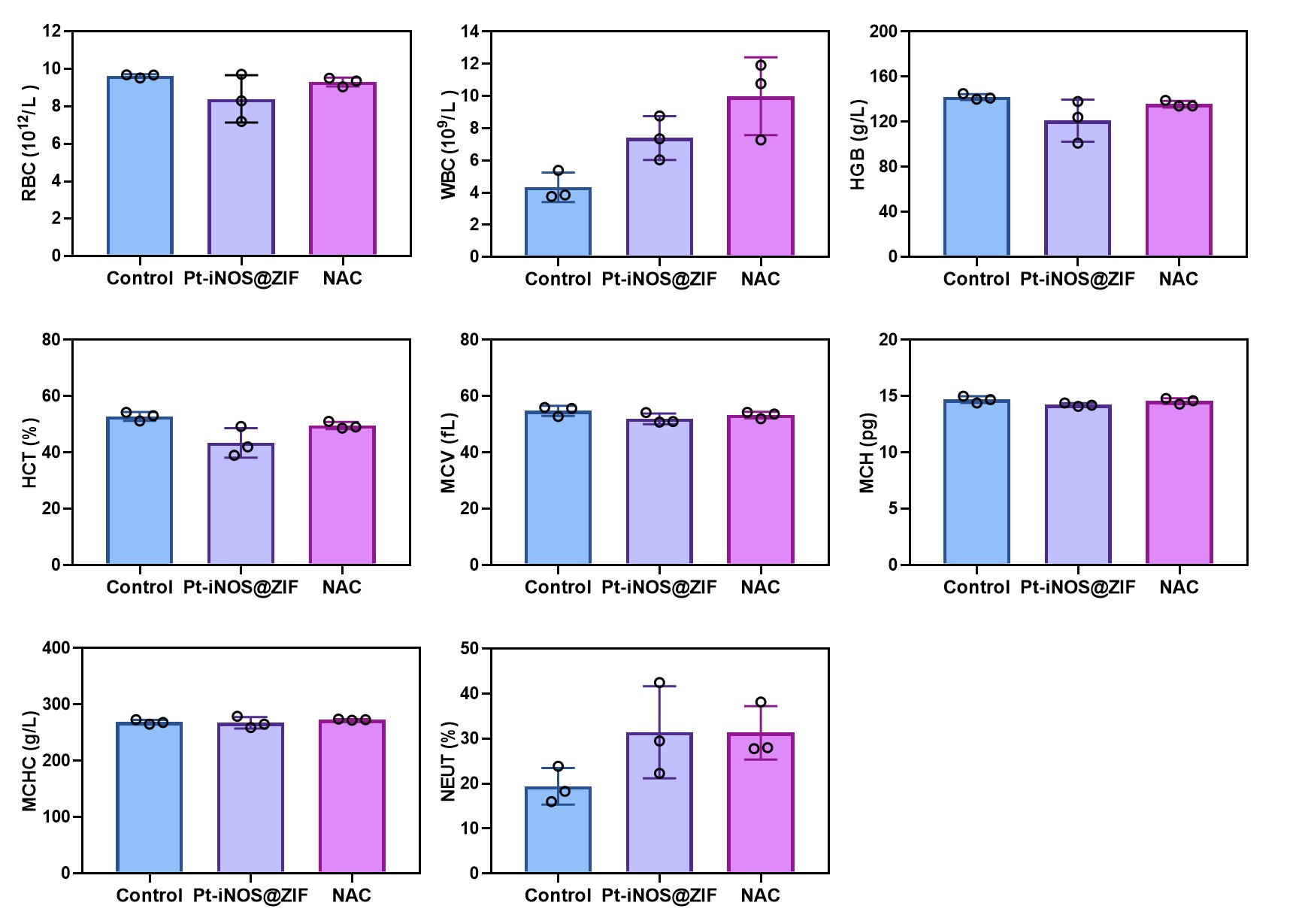


**Supplementary Fig. S17 Blood test for the evaluation of treatment effect.** To evaluate the physiological conditions of mice after the treatment, the complete blood panel data were assessed after the administration of Pt-iNOS@ZIF or NAC in IRI mice for 3 days (n = 3 biologically independent animals). red blood cells (RBC), white blood cells (WBC), hemoglobin (HGB), hematocrit (HCT), mean corpuscular volume (MCV), mean corpuscular hemoglobin (MCH), mean corpuscular hemoglobin concentration (MCHC), neutrophils (NEUT). Data are presented as means ± s.d.


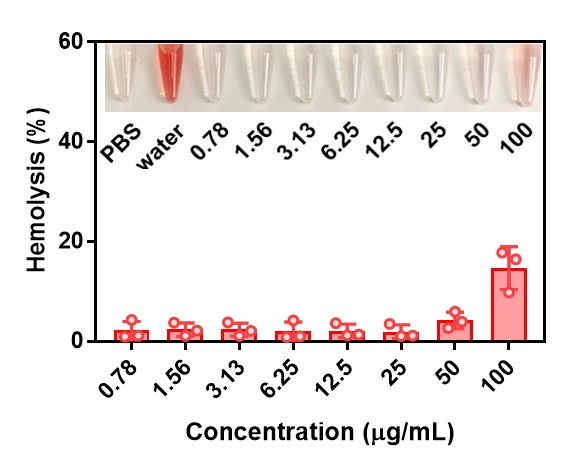


**Supplementary Fig. S18 Hemolysis test of Pt-iNOS@ZIF.** Red blood cells (RBCs) were isolated by centrifugation and diluted with PBS. 1 mL of RBC suspension was mixed with 0.2 mL of Pt-iNOS@ZIF at various concentrations (n = 3 biologically independent samples). Samples were centrifuged after 3 h incubation, and the absorbance of supernatants at 540 nm was recorded. The background was subtracted by control experiments and the percentages of hemolysis were calculated and plotted.

**
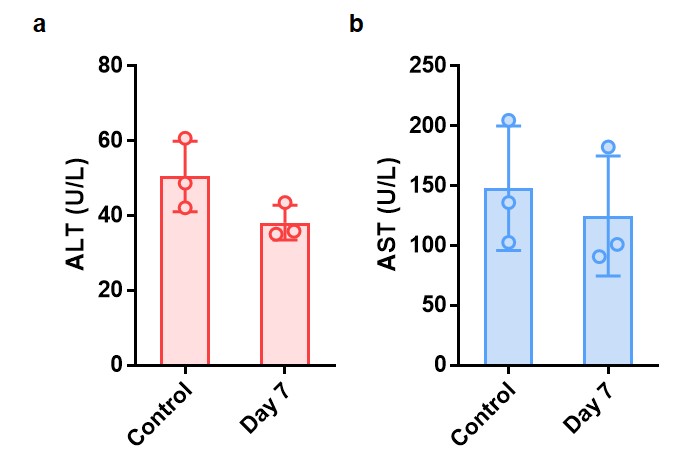
**

**Supplementary Fig. S19 Profiles of liver functions in healthy mice after injection with Pt-iNOS@ZIF for 7 days.** To evaluate the biocompatibility of nanoparticles, liver functions were assessed in healthy mice after intravenous injection of Pt-iNOS@ZIF for 7 days. Liver functions of untreated mice were also evaluated as control (n = 3 biologically independent animals).


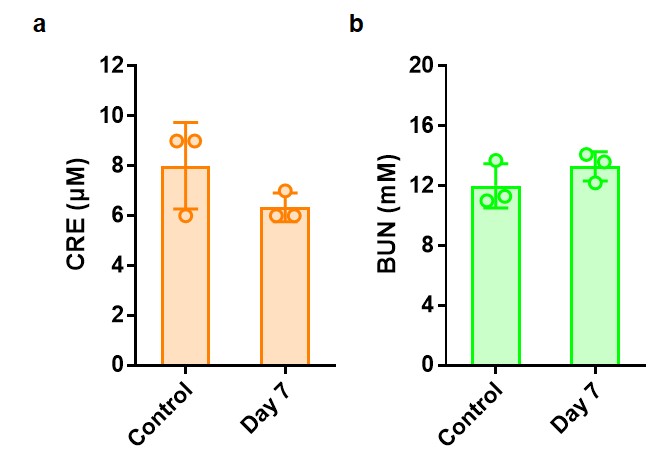


**Supplementary Fig. S20 Profiles of kidney functions in healthy mice after injection with Pt-iNOS@ZIF for 7 days.** To evaluate the biocompatibility of nanoparticles, kidney functions were assessed in healthy mice after intravenous injection of Pt-iNOS@ZIF for 7 days. Kidney functions of untreated mice were also evaluated as control n = 3 biologically independent animals). CRE: Creatinine, BUN: Blood urea nitrogen.


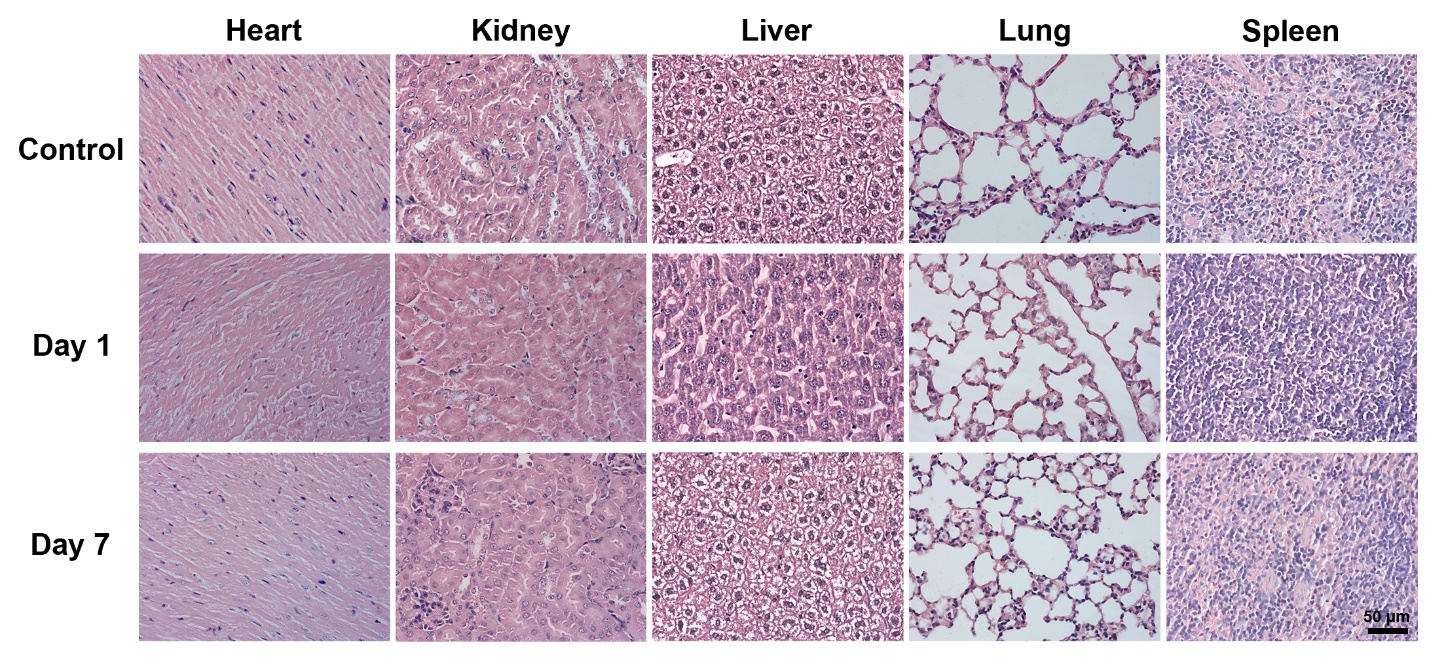


**Supplementary Fig. S21 H&E staining images of major organs in healthy mice.** To evaluate the safety of nanoparticles, major organs (heart, liver, spleen, lung and kidneys) were harvested at 24 h and 7 days for hematoxylin and eosin (H&E) staining. Experiments were performed three times with similar results


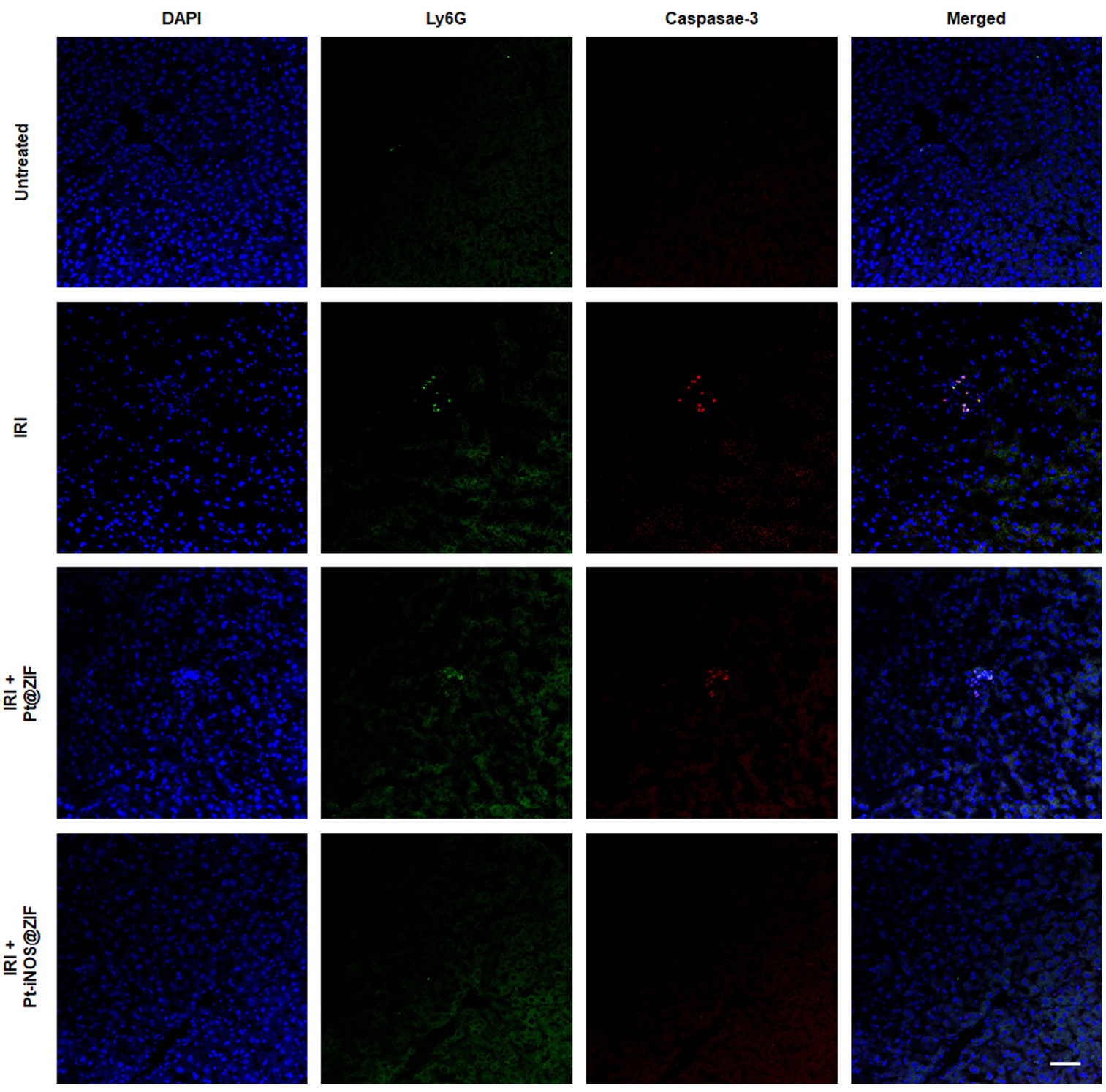


**Supplementary Fig. S22 Representative immunofluorescence staining on liver tissues.** images of immunofluorescence staining with various treatments by using DAPI (blue) for nuclear staining, anti-Ly6G antibody (green) as neutrophil marker and anti-caspase-3 antibody (red) as a cell apoptosis marker. Scale bar: 50 µm. Experiments were performed three times with similar results

**Supplementary Table 1**. The biodistribution analysis in major organs

| Organs | Heart | Liver | Spleen | Lung | Kidney |
| --- | --- | --- | --- | --- | --- |
| %ID | 0.55±0.12 | 22.84±3.65 | 1.94±0.60 | 1.09±0.26 | 3.02±0.64 |
| %ID g^-1^ | 5.54±1.25 | 22.84±3.65 | 9.72±2.99 | 5.47±1.28 | 10.06±2.14 |
| Weight (g) | 0.1 | 1 | 0.2 | 0.2 | 0.3 |

**Supplementary Table 2.** Quantification of immunofluorescence staining in Fig. 6c

| Group | Untreated | IRI | IRI + Pt@ZIF | IRI + Pt-iNOS@ZIF |
| --- | --- | --- | --- | --- |
| Ly6G | 2.134 | 2.596 | 2.341 | 1.875 |
| Caspase 3 | 1.887 | 2.562 | 2.077 | 1.698 |

The mean fluorescence intensity values were calculated by Image J software.

**Supplementary Table 3.** Primer information for mouse

| Gene | Forward | Reverse |
| --- | --- | --- |
| IL-1α | CCGTGTTGCTGAAGGAGTTG | GTGCACCCGACTTTGTTCTT |
| IL-1β | GCCCATCCTCTGTGACTCAT | TCAGCTCATATGGGTCCGAC |
| INF-γ | AGGATGGTGACATGAAAATCCTG | GAGCTCATTGAATGCTTGGC |
| IL-12α | CTCCTGTGGGAGAAGCAGAC | CAGATAGCCCATCACCCTGT |
| TNF-α | AGCCGATGGGTTGTACCTTG | ATAGCAAATCGGCTGACGGT |
| IL-6 | CACGGCCTTCCCTACTTCAC | TGCAAGTGCATCATCGTTGT |
| GAPDH | GGTGAGGGTCGGTGTGAACG | CTCGCTCCTGGAAGATGGTG |
