# Supplementary material for: Protective effect of platinum nano-antioxidant and nitric oxide against hepatic ischemia-reperfusion injury
Source: Nat Commun. 2022 May 6;13:2513. doi: 10.1038/s41467-022-29772-w (PMC9076604; doi:10.1038/s41467-022-29772-w)
Supplement: Supplementary file 4 — Reporting Summary [file 41467_2022_29772_MOESM4_ESM.pdf]

nature portfolio

Corresponding author(s): Xiaoyuan Chen
Last updated by author(s): 2022/02/23

Reporting Summary

Nature Portfolio wishes to improve the reproducibility of the work that we publish. This form provides structure for consistency and transparency in reporting. For further information on Nature Portfolio policies, see our Editorial Policies and the Editorial Policy Checklist.

Statistics

For all statistical analyses, confirm that the following items are present in the figure legend, table legend, main text, or Methods section.

n/a	Confirmed	
☐	☒	The exact sample size (*n*) for each experimental group/condition, given as a discrete number and unit of measurement
☐	☒	A statement on whether measurements were taken from distinct samples or whether the same sample was measured repeatedly
☐	☒	The statistical test(s) used AND whether they are one- or two-sided<br>*Only common tests should be described solely by name; describe more complex techniques in the Methods section.*
☒	☐	A description of all covariates tested
☒	☐	A description of any assumptions or corrections, such as tests of normality and adjustment for multiple comparisons
☐	☒	A full description of the statistical parameters including central tendency (e.g. means) or other basic estimates (e.g. regression coefficient) AND variation (e.g. standard deviation) or associated estimates of uncertainty (e.g. confidence intervals)
☐	☒	For null hypothesis testing, the test statistic (e.g. *F*, *t*, *r*) with confidence intervals, effect sizes, degrees of freedom and *P* value noted<br>*Give P values as exact values whenever suitable.*
☒	☐	For Bayesian analysis, information on the choice of priors and Markov chain Monte Carlo settings
☒	☐	For hierarchical and complex designs, identification of the appropriate level for tests and full reporting of outcomes
☒	☐	Estimates of effect sizes (e.g. Cohen's *d*, Pearson's *r*), indicating how they were calculated

Our web collection on statistics for biologists contains articles on many of the points above.

Software and code

Policy information about availability of computer code

Data collection	Transmission electron microscopy (TEM) images were acquired on a FEI Tecnai12 equipment with a voltage at 120 kV.<br>Dynamic light scattering measurements were performed at a SZ-100 nano particle analyzer (HORIBA Scientific).<br>UV-vis spectrum was recorded by a Shimadzu UV-2501 spectrophotometer using quartz cuvettes with an optical path of 1 cm.<br>The X-ray diffraction (XRD) pattern was recorded on a D8 Advance diffractometer (Bruker, Germany).<br>Flow cytometry data were collected using BD Accuri C6 Plus (BD Biosciences) and the equipped BD CSampler software.<br>Tissue and tumor samples for eosin (H&E) staining were observed using a BX41 bright field microscopy (Olympus).<br>Fluorescence images were acquired by confocal laser scanning microscopy (Zeiss LSM 780, Carl Zeiss).
Data analysis	Origin 2015, GraphPad Prism 7.0, Flow cytometry: FlowJo V10, Excel 2019,Chemdraw 2019 for windows.

For manuscripts utilizing custom algorithms or software that are central to the research but not yet described in published literature, software must be made available to editors and reviewers. We strongly encourage code deposition in a community repository (e.g. GitHub). See the Nature Portfolio guidelines for submitting code & software for further information.

Data

Policy information about availability of data

All manuscripts must include a data availability statement. This statement should provide the following information, where applicable:

- Accession codes, unique identifiers, or web links for publicly available datasets
- A description of any restrictions on data availability
- For clinical datasets or third party data, please ensure that the statement adheres to our policy

The authors declare that the experimental data supporting the findings of this study are available within the article and the Supplementary Information Files. Extra data are available corresponding author upon reasonable request. Source data are provided with this paper.

Field-specific reporting

Please select the one below that is the best fit for your research. If you are not sure, read the appropriate sections before making your selection.

☒ Life sciences ☐ Behavioural & social sciences ☐ Ecological, evolutionary & environmental sciences

For a reference copy of the document with all sections, see nature.com/documents/nr-reporting-summary-flat.pdf

Life sciences study design

All studies must disclose on these points even when the disclosure is negative.

Sample size	No statistical method was used to predetermine the sample size. The sample sizes were determined as minimal to lower the cost and be sufficient to obtain statistically significant difference between experimental groups (n=3-5).For property measurement experiments, samples were prepared and tested at least twice. For in vivo studies, each group contains at least 3 for evaluating the statistical significance. Fig. 5a-5f, n=3. Fig. 6a-6b, n=3. Fig. 7, n=3. Fig.S14, n=3.
Data exclusions	No data was excluded from the analysis.
Replication	All samples were replicated independently for 2-3 times with similar results. For each experiment the statistical analysis is indicated in the figure legends. All attempts at replication were successful.
Randomization	The samples were randomly grouped
Blinding	The investigators were not blinded to group allocation during data collection and analysis. Analyses in the animal experiments were based on measurements acquired and mostly performed by the same investigator.

Reporting for specific materials, systems and methods

We require information from authors about some types of materials, experimental systems and methods used in many studies. Here, indicate whether each material, system or method listed is relevant to your study. If you are not sure if a list item applies to your research, read the appropriate section before selecting a response.

Materials & experimental systems

n/a	Involved in the study	
☐	☒	Antibodies
☐	☒	Eukaryotic cell lines
☒	☐	Palaeontology and archaeology
☐	☒	Animals and other organisms
☒	☐	Human research participants
☒	☐	Clinical data
☒	☐	Dual use research of concern

Methods

n/a	Involved in the study	
☒	☐	ChIP-seq
☐	☒	Flow cytometry
☒	☐	MRI-based neuroimaging

Antibodies

Antibodies used	F4/80 Monoclonal Antibody (BM8), FITC, eBioscience (11-4801-85), Thermo Fisher Scientific. (1:100)<br>Alexa Fluor® 594 anti-mouse CD31 Antibody (Biolegend, 102520, 1:100)<br>Anti-Caspase-3 antibody (Abcam, ab13847, 1:200)<br>Goat Anti-Rabbit IgG H&L (Alexa Fluor 594) (Abcam, ab150080, 1:200)<br>anti-mouse Ly6G/Ly6C (Gr-1) (BioXcel, BE0075, 1:200<br>Goat Anti-Rat IgG H&L (Alexa Fluor 488) (Abcam, ab150157, 1:200)

Validation	All antibodies were verified by the supplier. The quality test data was showed on the manufactures' websites as following<br>F4/80 Monoclonal Antibody (BM8), FITC,<br>https://www.thermofisher.com/antibody/product/F4-80-Antibody-clone-BM8-Monoclonal/11-4801-85<br>Alexa Fluor® 594 anti-mouse CD31 Antibody<br>https://www.biolegend.com/ja-jp/products/alexa-fluor-594-anti-mouse-cd31-antibody-9633?GroupID=BLG10559<br>Anti-Caspase-3 antibody<br>https://www.abcam.com/caspase-3-antibody-ab13847.html<br>Goat Anti-Rabbit IgG H&L (Alexa Fluor 594)<br>https://www.abcam.com/goat-rabbit-igg-hl-alexa-fluor-594-ab150080.html<br>anti-mouse Ly6G/Ly6C (Gr-1) (BioXcel, BE0075<br>https://bxcell.com/product/m-ly-6g-2/<br>Goat Anti-Rat IgG H&L (Alexa Fluor 488)<br>https://www.abcam.cn/goat-rat-igg-hl-alexa-fluor-488-ab150157.html

Eukaryotic cell lines

Policy information about cell lines

Cell line source(s)	FL83B, HEK 293, Raw 264.7 cell lines were purchased from American Type Culture Collection (ATCC), Kuppfer cells were purchased from Thermal Fisher Scientific.
Authentication	Cells were only authenticated by the morphology.
Mycoplasma contamination	All cells were tested to be free of mycoplasma contamination.
Commonly misidentified lines<br>(See ICLAC register)	None.

Animals and other organisms

Policy information about studies involving animals; ARRIVE guidelines recommended for reporting animal research

Laboratory animals	Male C57BL/6 mice (6-8 weeks old) were received from Zhejiang Experimental Animal Center, China. The animals were hosted in equipped animal facility with ambient temperature of 22°C and humidity at 30%-70%, under a dark/light cycle of 12 h.
Wild animals	This study did not involve wild animals.
Field-collected samples	This study did not involve sample collected from the field.
Ethics oversight	All animal experiments were conducted according to the Guide of Animal Ethics Committee of Shanghai Skin Disease Hospital and the Animal Ethical and Welfare Committee of Shenzhen University (AEWC-SZU).

Note that full information on the approval of the study protocol must also be provided in the manuscript.

Flow Cytometry

Plots

Confirm that:

☒ The axis labels state the marker and fluorochrome used (e.g. CD4-FITC).

☒ The axis scales are clearly visible. Include numbers along axes only for bottom left plot of group (a 'group' is an analysis of identical markers).

☒ All plots are contour plots with outliers or pseudocolor plots.

☒ A numerical value for number of cells or percentage (with statistics) is provided.

Methodology

Sample preparation	IIntracellular ROS levels were measured using 2',7'-dichlorofluorescein diacetate (DCFH-DA) as the ROS indicator and NO levles were measured using nitric oxide indicators (DAF FM Diacetate). 8 μg/mL NPs were added into cells and cultured for 2 h, followed by the treatment with H2O2 (1 mM) and incubated for another 4 h at 37 °C. Then, the cells were stained with DCFH-DA (10 μM) or DAF probe (5 μM) for 30 min. After removing the excessive probe and debris by centrigugation twice, ROS and NO levels were analyzed by flow cytometry.
Instrument	BD Accuri C6 Plus
Software	FlowJo V10
Cell population abundance	The purity of post-sort fractions is regularly measured by the software. The fractions were around 50-70%.

Gating strategy

A forward-scatter/side-scatter gate was used to gate on cells.

☒ Tick this box to confirm that a figure exemplifying the gating strategy is provided in the Supplementary Information.
